# Metabolic control of daily locomotor activity mediated by *tachykinin* in *Drosophila*

**DOI:** 10.1038/s42003-021-02219-6

**Published:** 2021-06-07

**Authors:** Sang Hyuk Lee, Eunjoo Cho, Sung-Eun Yoon, Youngjoon Kim, Eun Young Kim

**Affiliations:** 1 Department of Biomedical Sciences, Ajou University Graduate School of Medicine, 164 Worldcup-ro, Suwon, Kyunggi-do Republic of Korea; 2 Department of Brain Science, Ajou University Medical Center, 164 Worldcup-ro, Suwon, Kyunggi-do Republic of Korea; 3 Korea Drosophila Resource Center, GIST, Oryong-dong, Buk-gu, Gwangju, Republic of Korea; 4 School of Life Sciences, Gwangju Institute of Science and Technology, 123 Cheomdangwagi-ro, Buk-gu, Gwangju, Republic of Korea

**Keywords:** Behavioural genetics, Circadian regulation

## Abstract

Metabolism influences locomotor behaviors, but the understanding of neural curcuit control for that is limited. Under standard light-dark cycles, *Drosophila* exhibits bimodal morning (M) and evening (E) locomotor activities that are controlled by clock neurons. Here, we showed that a high-nutrient diet progressively extended M activity but not E activity. *Drosophila tachykinin (DTk)* and *Tachykinin-like receptor at 86C (TkR86C)*-mediated signaling was required for the extension of M activity. DTk neurons were anatomically and functionally connected to the posterior dorsal neuron 1s (DN1_p_s) in the clock neuronal network. The activation of DTk neurons reduced intracellular Ca^2+^ levels in DN1_p_s suggesting an inhibitory connection. The contacts between DN1_p_s and DTk neurons increased gradually over time in flies fed a high-sucrose diet, consistent with the locomotor behavior. DN1_p_s have been implicated in integrating environmental sensory inputs (e.g., light and temperature) to control daily locomotor behavior. This study revealed that DN1_p_s also coordinated nutrient information through *DTk* signaling to shape daily locomotor behavior.

## Abstract (web summary)

Lee and colleagues report the effect of a high-sucrose diet on *Drosophila* locomotor activity via *DTk*-*TkR86C* neuropeptide signalling. This signalling pattern appears to involve a circadian element, with pacemaker neuron involvement having a possible time-of-day effect on locomotor behaviour.

## Introduction

The circadian clock system allows living organisms to anticipate environmental changes that are driven by the earth’s daily rotation, resulting in ~24-h rhythms in behavior and physiology. In animals, the cell-autonomous circadian clocks are organized into the master clock, residing in the brain, and peripheral clocks located throughout the body. The master clock is reset by external timing signals called zeitgebers, which in turn synchronize peripheral clocks through innervation and humoral signals^1,2^. The molecular mechanism controlling the circadian clock is a cell-autonomous transcriptional–translational feedback loop comprising the core clock genes^3,4^.

While the most potent zeitgeber is light, food also influences the circadian clock system^5–7^. Notably, timed-restricted feeding drives animal’s food-anticipatory activity^8^ and resets peripheral clocks, independent of master clocks^6,9^. Also, food content modulates rhythmic behaviors. In mice, a high-fat diet (HFD) reduces the rhythmicity and lengthens the periods of activity^10^, and a high-fat /high-salt diet reduces locomotor activity^11^. While metabolic control of the cell’s molecular clock has been extensively studied^12^, the effect of food on the neural circuit control of daily locomotor activity is not well understood. The fruit fly, *Drosophila melanogaster*, provides a genetically tractable model system to study fundamental aspects of the circadian clock and metabolism that are shared with mammals^3,13,14^.

In light–dark cycle, *Drosophila* exhibits bimodal patterns of locomotor activity with morning (M) peak and evening (E) peak, separated by a siesta. The flies' rhythmic locomotor activity profile is determined by the circadian neuron network located in the lateral and dorsal regions of the brain^15^. Large and small lateral ventral neurons (lLN_v_ and sLN_v_) called M oscillators control M activity, and lateral dorsal neurons (LN_d_) and a fifth sLN_v_ neuron called E oscillators control E activity^16–18^. Posterior dorsal neuron 1s (DN1_p_s) can control both M and E activities and integrate environmental stimuli, such as light and temperature, for locomotor regulation^19–23^.

Neuropeptides which control many aspects of behavior and physiology^24,25^ play a role in rhythmic locomotor activity. The pigment dispersing factor (PDF) released by LN_v_s synchronizes the clock neuron network and determines the anticipatory M activity and the phase of E activity. In addition, neuropeptide F (NPF), short neuropeptide F (sNPF), and the ion transport peptide (ITP) contribute to rhythmicity^26–28^. Diuretic hormone 31 (DH31) awakens flies in the early morning^29^, while Diuretic hormone 44 (DH44), expressed in the cells of neuroendocrine pars intercerebralis (PI), functions as an output molecule to communicate with the downstream locomotor center^30^. Neuropeptide leucokinin (LK), expressed in the lateral horn (LHLK), controls rhythmicity and levels of locomotor activity^31^. Some neuropeptides regulate both locomotor activity and metabolism; sNPF and NPF promote feeding and sleep^32,33^. After nutrient depletion, NPF signaling promotes feeding and suppresses sleep via independent circuits^34^. Conversely, allatostatin A suppresses feeding but promotes sleep^35^. Insulin-like hormone peptides (Ilps, an ortholog of mammalian insulin and insulin-like growth factor), which control metabolic homeostasis, regulate age-dependent sleep fragmentation^36^ and sleep depth in starved animals^37^. The neuropeptide SIFamide that is expressed in a subset of PI cells is required for rhythms of both locomotor activity and feeding/fasting^30,38^.

How dietary nutrient affects *Drosophila* locomotor behavior has been studied. A high-sucrose diet (HSD) reduces total sleep^39^ or alters the timing of sleep^40^. Siesta begins slightly later in *Drosophila* fed a HSD than in flies fed a low-sucrose diet (LSD)^40^. Sleep analysis in flies is based on measuring the duration of inactivity hence the delayed siesta onset associated with a HSD could instead reflect a lengthened period of M activity, but Linford et al. did not discuss M activity in detail^40^. Flies reared on a HFD increase their total sleep and bouts of sleep, together with reduced lifespan and fecundity mediated by increased expression of adipokinetic hormone (AKH)^41^. Although the locomotor assay is used for sleep studies, the molecular and neural mechanisms that control sleep versus activity are clearly separable. However, the effects of diet on daily locomotor activity and their underlying neural mechanisms are unknown.

In this study, we examined *D. melanogaster’s* daily locomotor activity in high-nutrient conditions and investigated underlying neuropeptidergic control mechanisms. A HSD or a HFD extended M activity, but not E activity. *DTk-* and *TkR86C*-mediated signaling were required for the extended M activity. *DTk*-expressing neurons were anatomically and functionally connected to DN1_p_s. The contacts between DN1_p_s- and *DTk*-expressing neurons gradually increased over time in flies fed a HSD, which is consistent with the locomotor activity behavior. Collectively, these results indicated that in addition to the role of integrating temperature signals into the circadian clock, DN1_p_s integrated nutrient information through *DTk* signaling and controlled *Drosophila* locomotor behavior in a nutrient state-dependent manner.

## Results

### HSD extended morning activity but not evening activity

**Fig. 1:**
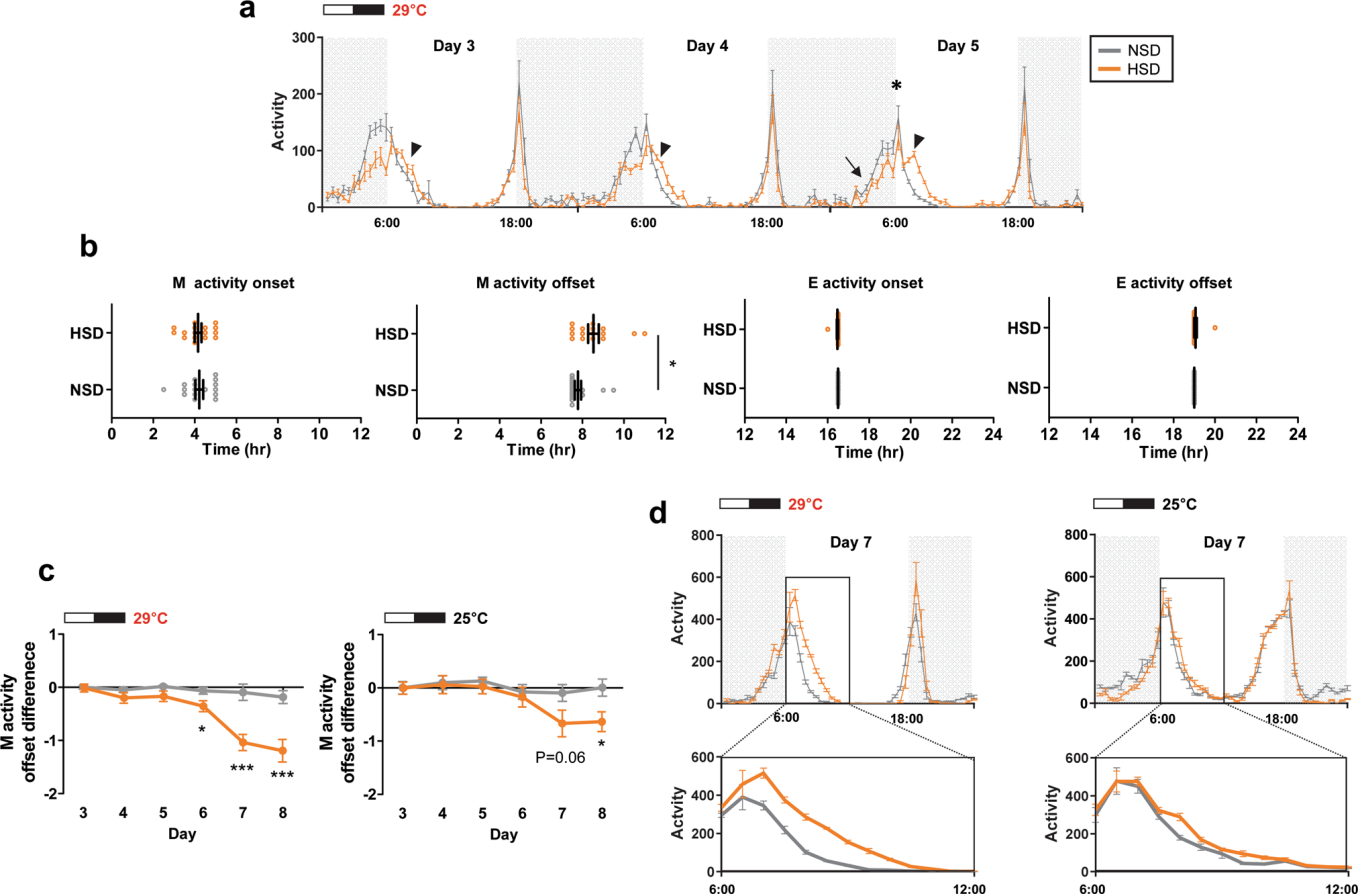
Fly M activity but not E activity offset was extended in HSD. **a**, **b** *w*^1118^ fly locomotor activity was analyzed in normal sucrose diet (NSD) and high-sucrose diet (HSD) under a 12L:12D cycle at 29 °C. **a** Daily activity profiles from day 3 to day 5 are shown. Arrow indicates anticipatory activity and asterisk indicates the startle response. M activity, but not E activity, was extended in flies fed a HSD (arrowhead). **b** M and E activity onset and offset times for individual *w*^1118^ flies on day 5 are shown. Bars indicate mean ± SEM values (*n* = 28–31). Statistically significant differences in the onset or offset between NSD and HSD (independent *t* test): *P < 0.05. **c**, **d** *w*^1118^ fly locomotor activity was analyzed in NSD and HSD under 12L:12D cycle at 29 °C and 25 °C. Daily activity profiles for days 3–8 are in Supplementary Fig. 2. **c** M activity offsets of individual *w*^1118^ flies were obtained and the differences versus day 3 are shown. The M activity offset in HSD was progressively delayed at 29 °C and 25 °C. Values indicate mean ± SEM (*n* = 30–37). Statistically significant differences in the M activity offset between NSD and HSD at each day (independent *t* test): *P < 0.05; ***P < 0.001. **d** Daily activity profiles on day 7 at 29 °C and 25 °C are shown. Lower panels show a magnified image of the boxed region in the upper panel. The extent of M activity offset delay was greater at 29 °C than at 25 °C.

To evaluate how nutrient concentration affected fly daily locomotor behavior, we analyzed *Drosophila melanogaster* activity in normal concentration (5% sucrose, normal sucrose diet, NSD) or high concentration (30% sucrose, high sucrose diet, HSD) sucrose-containing food in a 12-h light/12-h dark (12L:12D) condition at 29 °C. Flies exhibited bimodal M and E activities around light on/off transition. The control *w*^1118^ flies fed on a NSD or a HSD showed differences in the M activity but not the E activity (Fig. 1a). Anticipatory activities controlled by the circadian clock (arrow, Fig. 1a) and the startle responses that occurred immediately after the light on/off transitions (asterisk, Fig. 1a) were not largely different under the two diet conditions^42^. However, while M activity in NSD was sharply reduced following the startle response M activity was extended in HSD (arrowhead, Fig. 1a). To quantitate this behavior for the two diet conditions, we compared locomotor activity onset and offset times (Fig. 1b). Activity onset indicated the largest 1-h increase before the light transition, while activity offset indicated the largest 1-h decrease after the light transition. As expected from the activity pattern (Fig. 1a), while the M activity onset, the E activity onset, and E activity offset were the same between the two diets, the M activity offset was delayed about 1 h, on average, in flies fed a HSD on day 5 compared to the M activity offset in flies fed a NSD (Fig. 1b). To test whether this effect was specific to sucrose, we measured fly locomotor activity in HFD containing 20% coconut oil with NSD. Because coconut oil melted at high temperatures, we performed the behavior analysis at 25 °C. Compared with the NSD, M activity was extended after the startle response in both the HSD and HFD, but E activity was not different in any diets (Supplementary Fig. 1a). Activity offset was delayed only in the mornings in HFD and HSD conditions (Supplementary Fig. 1b). We also noted that the effect of a high-nutrient diet on M activity was enhanced over time (Fig. 1a and Supplementary Fig. 2). M activity offset showed a delay each day until day 7 after which the delay was maintained (Fig. 1c, d). The HSD-induced M activity offset delay was somewhat reduced at 25 °C compared to 29 °C (Fig. 1d and Supplementary Fig. 2). Collectively, M activity was extended after the startle response when the nutrient content was high, and high-temperature augmented this effect.

**Fig. 2:**
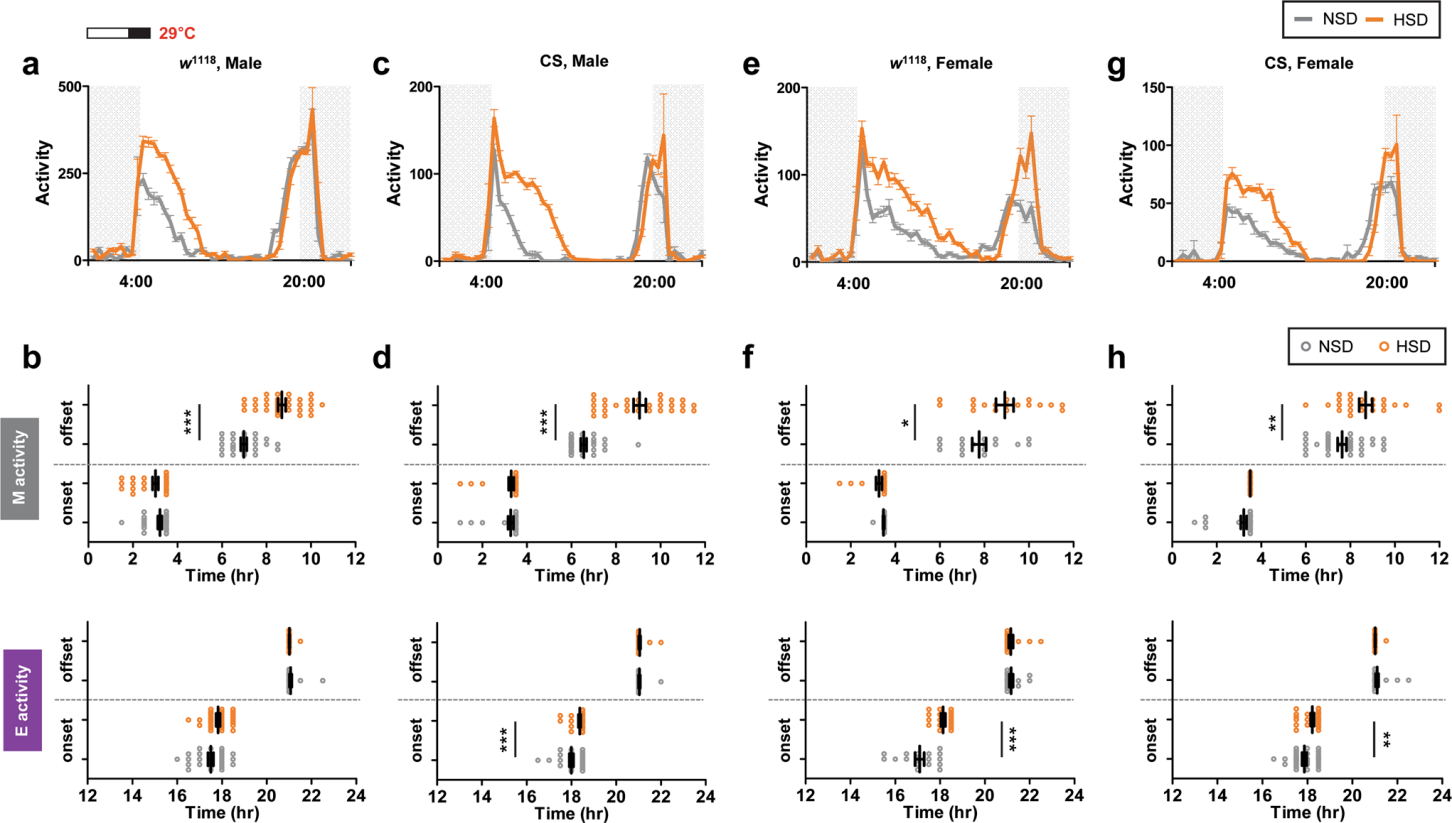
The effect of a HSD on M activity offset was observed in other genotypes of control flies and in females. **a**, **c**, **e**, **g** Locomotor activities of *w*^1118^ male (**a**) and female (**e**), Canton S (CS) male (**c**), and female (**g**) flies locomotor activities were analyzed in NSD and HSD conditions under a 16L:8D cycle at 29 °C. Daily activity profiles of given genotypes of flies (denoted on top) on day 7 are shown. **b**, **d**, **f**, **h** M and E activity onset/offset of *w*^1118^ male (**b**), *w*^1118^ female (**f**), CS male (**d**), and CS female (**h**) flies on day 7 are plotted. Bars indicate mean ± SEM (*n* = 15–32). Statistically significant differences in the onset or offset between NSD and HSD conditions (independent *t* test): *P < 0.05, **P < 0.01, ***P < 0.001.

Since only the morning locomotor activity was extended in flies fed a HSD, we attempted to increase the phase relationship between the M and E activities by exposing flies to a long-day photoperiod, 16L:8D^43^. Extended M activity after the startle response was more prominent in the 16L:8D compared to 12L:12D (Figs. 1a and  2a). The extent of M activity offset delay was greater in the 16L:8D cycle compared to the 12L:12D, but M activity onset, E activity onset, and E activity offset were not altered in HSD (Fig. 2b). Therefore, we used a 16L:8D condition for the subsequent experiments and analyzed the activity profile at day 7 if not mentioned otherwise.

Wild-type flies, Canton S, also had extended M activity (Fig. 2c) and showed an M offset delay in a HSD (Fig. 2d). Next, to examine for gender differences, we analyzed female locomotor behavior and found the same HSD effect on M activity for both male and female flies (Fig. 2e–h). In some instances, the E activity onset was slightly delayed but not as consistently as the M activity offset. Taken together, these results indicated that M activity extension in high-nutrient conditions is a universal behavioral response of *Drosophila*.

### Neuropeptide tachykinin was required for M activity extension in flies on a HSD

**Fig. 3:**
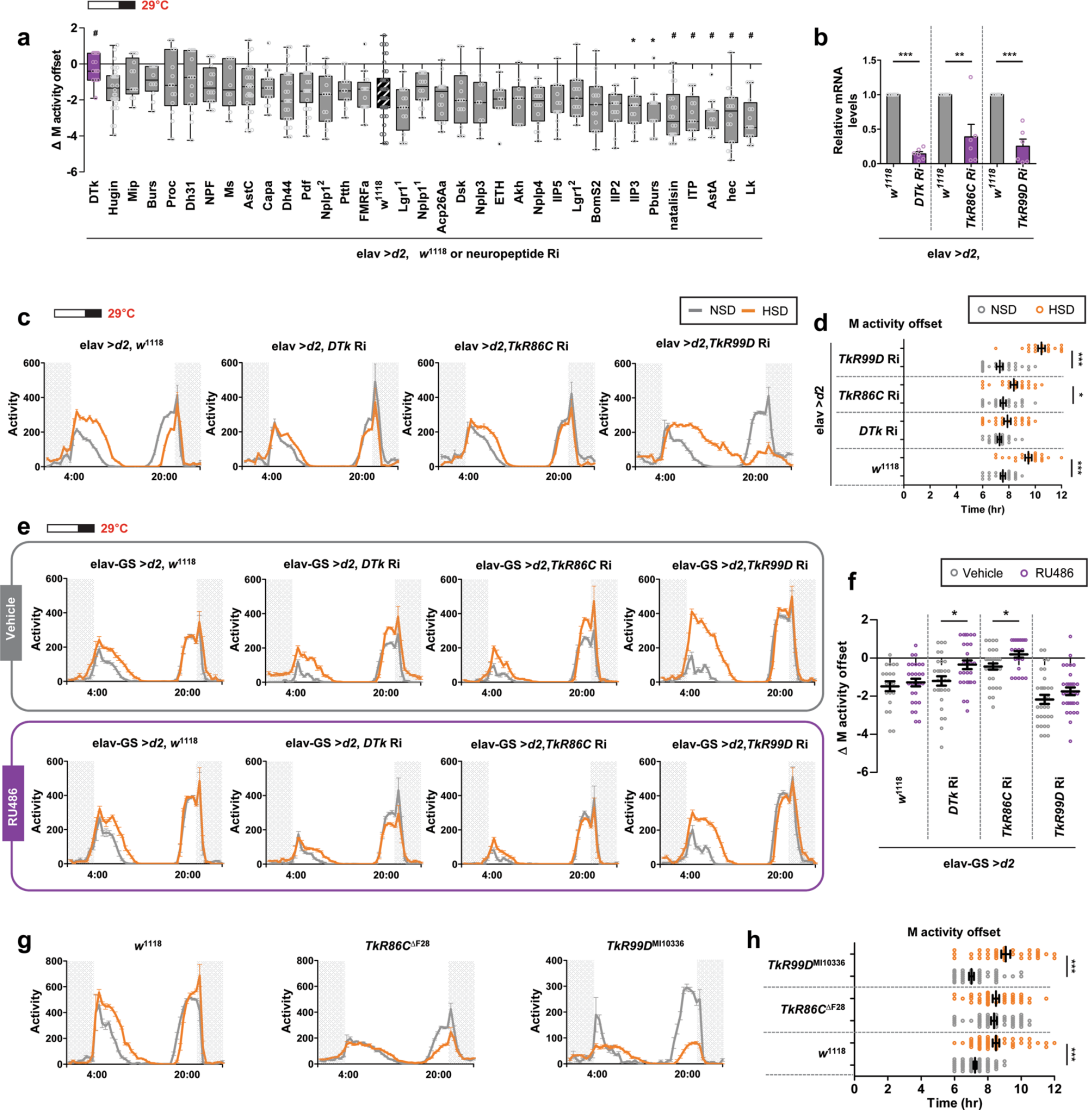
Neuropeptide DTk and the DTk receptor TkR86C were required for M activity extension in HSD. **a**–**d** *w*^1118^ flies or UAS-neuropeptide RNAi (denoted on bottom) flies were crossed with elav-Gal4, UAS-*dcr2* (elav > *d2*). **a** The locomotor activities of offspring were analyzed in NSD or a HSD under a 16L:8D cycle at 29 °C. Differences in average M activity offset between NSD and HSD (ΔM activity offset) on day 7 are shown. Control flies (hatched box;elav > *d2*, *w*^1118^) showed delayed M activity offset in HSD. The *DTk* knockdown flies (purple box; elav > *d2*, *DTk* Ri) showed little difference with a NSD versus a HSD (*n* = 9–16). Statistically significant differences in ΔM activity offset between the control and knockdown flies (independent *t* test): *P < 0.05, ^#^*P* < 0.01. **b** Flies with the indicated genotypes fed a NSD on a 16L:8D cycle at 29 °C were collected at ZT2. DTk, TkR86C, and TkR99D mRNA levels were quantified by qRT-PCR. The mRNA levels in the knockdown flies were normalized to the control (elav > *d2*, *w*^1118^) flies. Values indicate mean ± SEM from six independent experiments. Statistically significant differences in mRNA levels between control and knockdown flies (independent *t* test): **P < 0.01, ***P < 0.001. **c**–**h** The locomotor activities of each fly genotype (denoted above each graph) were analyzed in NSD and HSD under a 16L:8D cycle at 29 °C. **c**, **e**, **g** Daily activity profiles of flies on day 7 are shown. **d**, **h** The M activity offsets of individual flies on day 7 are shown. Bars indicate mean ± SEM (*n* = 21–62). Statistically significant differences in the average time between NSD and HSD (independent *t* test): *P < 0.05, ***P < 0.001. **f** ΔM activity offset on day 7 are shown. Statistically significant differences in ΔM activity offset between vehicle- and RU486-treated groups for each genotype of flies (independent *t* test): *P < 0.05.

Neuropeptides are small proteins that modulate many aspects of physiology and behavior, such as feeding and rhythmic locomotor behavior^24,25^. We searched for neuropeptides mediating the HSD effect on locomotor behavior using an RNAi screen via a binary Gal4/UAS system^44^. Neuron-specific elav-Gal4 driver flies were crossed with *w*^1118^ or UAS-neuropeptide RNAi flies, and the offspring were used as a control or knockdown flies, respectively. Thirty-four neuropeptide genes were tested, and the ΔM activity offset on day 7 was determined and compared to the control (elav > *dcr2, w*^1118^) (Fig. 3a). Eight neuropeptide knockdown flies had an enhanced HSD-associated response. The *DTk* knockdown flies showed only a reduced HSD-associated response. Downregulation of *DTk* mRNA in the heads of the knockdown flies was confirmed by qRT-PCR (Fig. 3b). Daily activity profiles and comparisons of the M activity offset in individual flies indicated that pan-neuronal knockdown of *DTk* reduced the HSD-associated effects on M activity (Fig. 3c, d and Supplementary Fig. 3). Two DTk receptor isoforms, *TkR86C* (CG6515; neurokinin receptor from *Drosophila*, NKD) and *TkR99D* (CG7887; *Drosophila* tachykinin receptor, DTkR) have been cloned in *Drosophila*^45–48^. Pan-neuronal knockdown of *TkR86C* but not *TkR99D*, confirmed by qRT-PCR (Fig. 3b), diminished the M activity extension in flies fed a HSD (Fig. 3c, d). Knockdown of *DTk* also diminished the effects of a HSD in standard 12L:12D conditions at 25 °C (Supplementary Fig. 4). We then used the drug-inducible pan-neuronal elav-GeneSwitch driver to determine whether knockdown of *DTk* or *TkR* in adults diminished the HSD effect^49^. Flies in which *DTk* or *TkR86C* expression was downregulated by ingestion of RU486 showed less effect of a HSD compared with the control vehicle-treated flies (Fig. 3e, f). Flies in which most of the first exon of *TkR86C* was deleted (*TkR86C*^ΔF28^) did not show M activity extension in a HSD (Figs. 3g, h)^50^. In contrast, a putative loss-of-function insertion mutation of *TkR99D*, *TkR99D*^MI10336^, did not affect the HSD-induced M activity offset delay (Fig. 3g, h). Interestingly, flies with loss-of-function mutations in *TkR86C* or *TkR99D* showed reduced E activity in HSD, suggesting that there might be a common regulatory role for these two receptors on E activity in flies fed a HSD.

*Tk* includes an evolutionarily well-conserved family of brain/gut neuropeptides that function as important neuromodulators in the central and peripheral nervous systems (reviewed in ref. ^51^). *DTk*s are also involved in various aspects of behavior and physiology, including locomotion^52,53^ and food-seeking behavior^50^. We tested the possibility that the downregulation of *DTk* or *TkR86C* affects feeding, thereby contributing to the reduced response in flies fed a HSD; however, food intake was similar in control, *DTk* and *TkR86C* knockdown flies consuming either diet (Supplementary Fig. 5). We also tested whether *DTk* signaling affects circadian rhythmicity behavior. Flies were entrained under the 12L:12D cycle followed by constant darkness, and their circadian locomotor behavior was analyzed in NSD or HSD (Supplementary Fig. 6). elav > *d*2, *DTk* Ri, elav > *d*2, *TkR86C* Ri, and elav > *d*2, *TkR99D* Ri flies exhibited a similar period and rhythmicity compared to control flies, indicating *DTk* signaling is not involved in regulating general circadian locomotor behavior. Nevertheless, all the fly genotypes showed a tendency toward a lengthened period and reduced robustness of rhythm in HSD, consistent with the previous mammalian study conducted in HFD^50^. In this behavior analysis, we also noted the M activity extension and offset delay in constant darkness; however different from LD cycle, E activity onset was slightly delayed in HSD. In constant darkness, the duration of the siesta decreased due to the absence of a strong, light-driven paradoxical masking effect^54–56^. Thus, the homeostatic drive to maintain a critical length of siesta might delay E activity onset. Taken together, these results revealed that neuropeptide *DTk* signaling via *TkR86C* specifically mediated the HSD-induced M activity extension in *Drosophila*.

### DTk levels were upregulated in DTk neurons in flies on a HSD

**Fig. 4:**
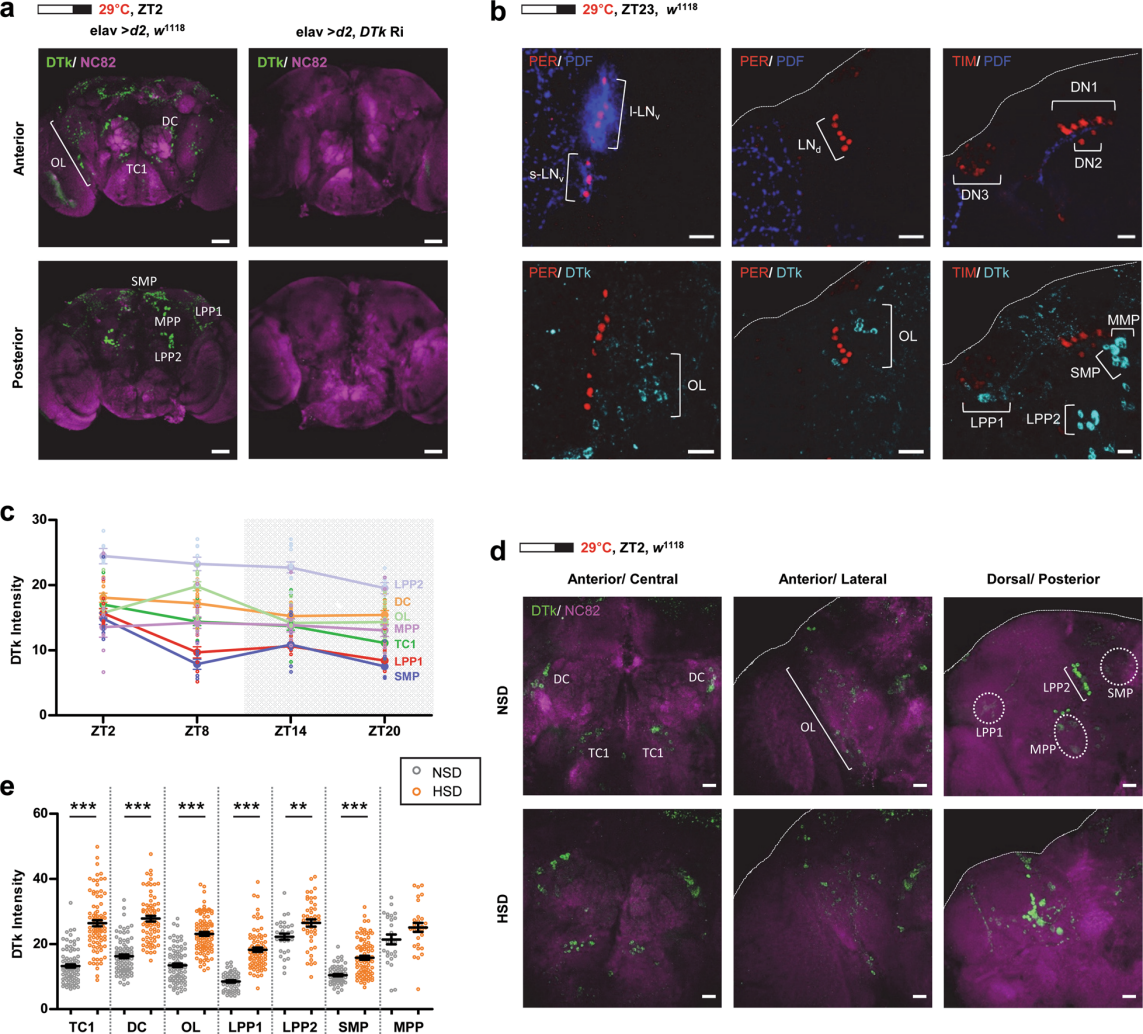
DTk levels were increased in DTk neurons in flies fed a HSD. **a** Flies of the indicated genotypes were maintained under 16L:8D cycle at 29 °C. Brains were dissected at ZT2 and stained with anti-DTk (green) and anti-NC82 (magenta) antibodies. In control flies (elav > *d2*, *w*^1118^), DTk-positive clusters were observed in DC, TC1, and in the OL on the anterior side. On the posterior side, DTk-positive clusters were observed in the SMP, LPP1, LPP2, and the MPP. DTk-positive clusters were absent in pan-neuronal DTk knockdown flies (elav > *d*2, *DTk* Ri). All scale bars represented 50 μm. **b** *w*^1118^ flies were maintained under a 16L:8D cycle at 29 °C. Brains were dissected at ZT23 and stained with anti-PER (red), anti-TIM (red), anti-PDF (blue), and anti-DTk (cyan blue) antibodies. DTk-positive neurons and clock neurons did not overlap but were in close proximity. All scale bars represented 20 μm. **c** *w*^1118^ flies were maintained with a NSD and 12L:12D cycle at 29 °C. Brains were dissected at each indicated time and stained with anti-DTk antibodies. DTk intensities of each cluster were quantified using ImageJ software and are shown. **d**, **e** *w*^1118^ flies were maintained with a NSD or HSD under a 16L:8D photoperiod at 29 °C. Brains were dissected on day 7, ZT2, and stained with anti-DTk (cyan blue) and anti-NC82 (magenta) antibodies. All scale bars represented 50 μm. **e** DTk intensities in each DTk-positive cluster were quantified using ImageJ software. Bars indicate mean ± SEM (*n* = 26–98). Statistically significant differences in the average intensity value between NSD and HSD (independent *t* test): **P < 0.01, ***P < 0.001.

To examine how *DTk* mediated HSD affects on M activity, we immunostained fly brains with newly raised DTk antibodies. Immunostaining of control fly brains revealed DTk-positive clusters consistent with previous study^51,57^. In the anterior region, deutocerebrum (DC), tritocerebrum (TC1), and optic lobe (OL) clusters were DTk-positive. In the posterior region, superior median protocerebrum (SMP), lateral posterior protocerebrum 1 (LPP1), lateral posterior protocerebrum 2 (LPP2), and median posterior protocerebrum (MPP) clusters were DTk-positive (Fig. 4a). elav-Gal4 driving *DTk* knockdown flies did not exhibit DTk staining, which verified the antibody specificity and the downregulation of *DTk* in the knockdown flies. Because the DTk-positive neurons were in the lateral and dorsal areas where clock neurons are located, we determined whether the DTk-positive neurons were clock neurons. Clock neurons (e.g., LN_v_s, LN_d_s, and DNs) labeled with anti-PERIOD (PER) or anti-TIMELESS (TIM) antibodies were contiguous with DTk-labeled neurons but did not overlap (Fig. 4b).

We then assessed DTk levels under different diets. We first examined DTk levels throughout the day in flies fed a NSD and found that levels were highest early in the morning (e.g., ZT2) in most DTk neuronal clusters (Fig. 4c). Flies were maintained in a NSD or HSD, and on day 7 brains were dissected at ZT2 and immunostained with DTk antibody (Fig. 4d). DTk staining intensities were higher in every DTk neuron in the brains from the flies fed a HSD compared with the NSD (Fig. 4e). The DTk signal increase was observed in the soma and the knockdown of *DTk* or *TkR* using RNAi attenuated the HSD effects; therefore, the enhanced staining intensity likely resulted from increased expression rather than inhibition of DTk release in HSD.

### DTk neurons and DN1_p_s were anatomically and functionally connected

**Fig. 5:**
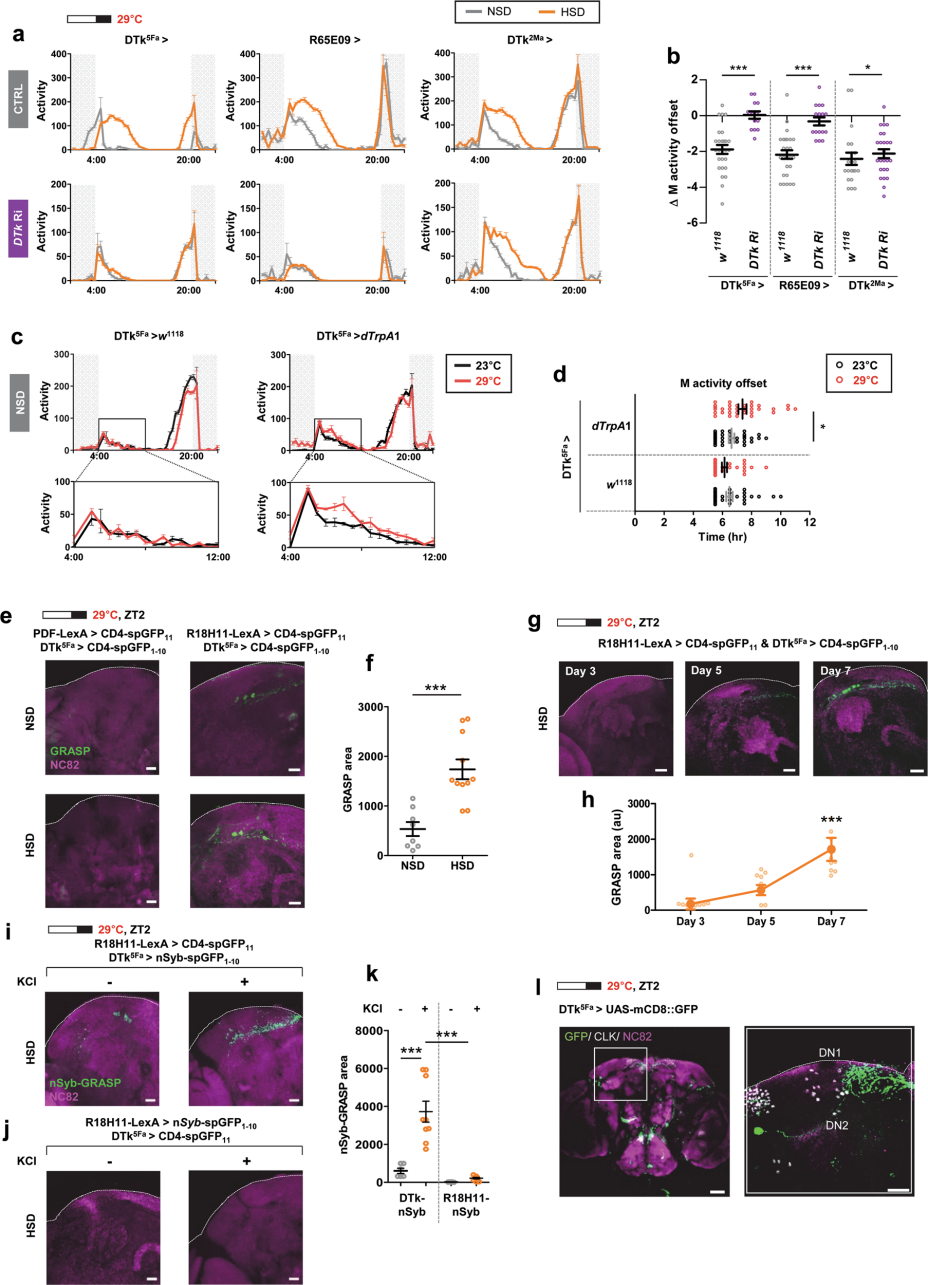
DTk neurons and DN1_P_s were anatomically and functionally connected. **a**, **b** Fly locomotor activity for *w*^1118^ (control) or UAS-*DTk* Ri (*DTk* Ri) driven by different DTk-Gal4 (DTk^5Fa^-Gal4, DTk^2Ma^-Gal4, and R65E09-Gal4) was analyzed in NSD and HSD under a 16L:8D cycle at 29 °C. **a** Daily activity profiles of flies on day 7 are shown. **b** ΔM activity offsets on day 7 are shown. *DTk* knockdown in DTk^5Fa^-Gal4, and 65E09-Gal4 active cells abolished the HSD effect, but not in the DTk^2Ma^-Gal4 active cells. (*n* = 14–26). Statistically significant differences in ΔM activity between control and *DTk* knockdown flies (independent *t* test): *P < 0.05, ***P < 0.001. **c**, **d** Flies were entrained in 16L:8D cycle at 23 °C for 7 days in NSD. The temperature was then elevated to 29 °C for 2 more days. **c** Daily activity profiles of flies on the last day at 23 °C (before activation) and on the 2nd day at 29 °C were overlaid. **d** M activity offset of individual flies on the last day at 23 °C and on the 2nd day after the temperature elevation to 29 °C to avoid temperature change-induced strong startle activity are shown. Bars indicate mean ± SEM (*n* = 32). Statistically significant differences in the average time between 23 and 29 °C (independent *t* test): *P < 0.05. **e**–**l** Flies of the indicated genotypes were maintained on a 16L:8D cycle at 29 °C. Brains were dissected at ZT2. **e**, **f** On day 7, GRASP-positive signals were produced between DN1_p_s in R18H11-LexA > LexAop-CD4-spGFP_11_ and DTk neurons in DTk^5Fa^ > UAS-CD4-spGFP_1-10_, but not between LN_v_s in PDF-LexA > LexAop-CD4-spGFP_11_ and DTk neurons in DTk^5Fa^ > UAS-CD4-spGFP_1-10_. GRASP signals were detected more broadly in flies fed a HSD. All scale bars represented 20 μm. **f** The areas showing GRASP signals were quantified using ImageJ software (*n* = 8–11). Statistically significant differences in GRASP area between NSD and HSD groups (independent *t* test): ***P < 0.001. **g**, **h** GRASP signals between DTk neurons in DTk^5Fa^ > UAS-CD4-spGFP_1-10_ and DN1_p_s in R18H11-LexA > LexAop-CD4-spGFP_11_ were analyzed on days 3, 5, and 7. All scale bars represented 20 μm. **h** The areas showing GRASP signals were quantified using ImageJ software. The GRASP areas were progressively increased over time in flies fed a HSD. Values indicate mean ± SEM (*n* = 8–9). Statistically significant differences in GRASP area (one-way ANOVA): ***P 0.001. **i**–**k** Flies of the indicated genotypes were maintained on a 16L:8D cycle at 29 °C. Brains were dissected at ZT2. On day 7, nSyb-GRASP-positive signals were produced between DN1_p_s in R18H11-LexA > LexAop-CD4-spGFP_11_ and DTk neurons in DTk^5Fa^ > UAS-nSyb-spGFP_1-10_ (**i**), but not between DN1_p_s in R18H11-LexA > LexAop-nSyb-spGFP_1-10_ and DTk neurons in DTk^5Fa^ > UAS-CD4-spGFP_11_ (**j**). Stronger nSyb-GRASP signals were detected when brains were exposed to KCl (final 70 mM, +) than to AHL (−). All GRASP- and nSyb-GRASP-positive signals represented endogenous GFP fluorescence. Brains were counter-stained with anti-NC82 (magenta) antibodies. All scale bars represented 20 μm. **k** The areas showing nSyb-GRASP-positive signals were quantified using ImageJ software (*n* = 6–9). Statistically significant differences in nSyb-GRASP area between NSD and HSD groups (independent *t* test): ***P < 0.001. **l** Flies of the indicated genotypes (denoted on top) were maintained on a 16L:8D cycle at 29 °C. Brains were dissected at ZT2 and stained with anti-GFP (green), anti-CLK (gray), and anti-NC82 (magenta) antibodies. The right panel shows magnified images of the boxed regions in the left panel. All scale bars represented 20 μm.

Instead of using elav-Gal4 to knock down *DTk* in a pan-neuronal manner, *DTk* was downregulated in limited groups of cells using three Gal4 lines, DTk^5Fa^-Gal4, DTk^2Ma^-Gal4, and R65E09-Gal4. DTk^5Fa^-Gal4 and DTk^2Ma^-Gal4 were generated in the collection of neuropeptide promoter-GAL4 strains^58,59^. R65E09-Gal4, a Janelia Gal4 line, is associated with the DTk promoter region^60,61^. While three DTk-Gal4 control flies exhibited M activity extension (Fig. 5a) and prominent M offset delays (Fig. 5b) in HSD, the knockdown of *DTk* using DTk^5Fa^-Gal4 or R65E09-Gal4, but not DTk^2Ma^-Gal4, abolished the HSD effects. Thus, DTk^5Fa^ and R65E09-Gal4 neurons mediated the HSD effects on M activity.

Next, we investigated whether acute manipulation of DTk neuronal activity affected M activity in NSD. We activated DTk neuron subsets by expressing warmth-activated cation channel, *dTrpA1*, which is inactive below 25 °C^62^. Because DTk^5Fa^-Gal4-driven *DTk* RNAi successfully suppressed the M activity extension, we used DTk^5Fa^-Gal4 for neuronal activity manipulation. Flies were entrained in a 16L:8D cycle at the non-permissive temperature of 23 °C for 7 days in NSD, and then the temperature was elevated to the permissive temperature of 29 °C for 2 more days. Locomotor activities and M activity offsets were compared on the last day at 23 °C and on the 2nd day after temperature elevation to avoid temperature change-induced strong startle activity. M activity in control flies (DTk^5Fa^ > *w*^1118^) was the same at 23 °C or 29 °C, indicating that the temperature increase alone did not affect M activity significantly. On the other hand, flies expressing *dTrpA1* (DTk^5Fa^ > *dTrpA1*) exhibited a small but obvious M activity increase at 29 °C (Fig. 5c). In control flies, temperature elevation alone induced M activity offset differences were not evident. However, for flies expressing *dTrpA1* (i.e., DTk neurons were activated by temperature elevation) there was a slight but statistically significant M activity offset delay (Fig. 5d). On the other hand, the warmth-induced activation of DTk^2Ma^–Gal4, which did not affect the HSD-associated behavior (Fig. 5a), did not increase M activity (Supplementary Fig. 7). Collectively, DTk neuron subgroup’s activity was involved in the HSD mediated M activity extension.

The M activity is controlled by sLN_v_s and DN1_p_s^19,22,30,63^, suggesting that DTk neurons might affect M activity by communicating with either sLN_v_s or DN1_p_s. To test this idea, we performed a GFP Reconstitution Across Synaptic Partners (GRASP) experiment to examine synaptic connections between two cells^64,65^ (Fig. 5e). We paired the Pdf-LexA (LN_v_s driver)^66^ or the R18H11-LexA (DN1_p_s driver)^29^ with DTk^5Fa^-Gal4 to express the split-GFP fragments, UAS-CD4::spGFP_1-10_ and LexAop-CD4::spGFP_11_. When the split-GFP fragments were in LN_v_s and DTk cells, no GFP signal was reconstituted. In contrast, high GFP signals were observed when split-GFP fragments were expressed by R18H11-LexA and DTk^5Fa^-Gal4. We found reconstituted GFP signals in the soma and nearby neurites in the DN1 region, indicating that DTk neurons were in physical contact with DN1_p_s but not with LN_v_s. We also found that reconstituted GFP-labeled neurites were greatly increased in HSD. We quantified this by measuring the GRASP signal area, and the results indicated that physical contacts increased between DTk neurons and DN1_p_s in flies fed a HSD (Fig. 5f). The M activity extension behavior was progressively enhanced over time (Fig. 1c and Supplementary Fig. 2). Intriguingly, the GRASP area between DTk neurons and DN1_p_s gradually increased over time for flies fed a HSD (Fig. 5g, h). These results support the idea that the HSD-induced M activity extension required plasticity in synapses between the DTk neurons and DN1_p_s.

Next, we used a modified GRASP technique to determine whether GRASP signals between DN1_p_s and DTk neurons resulted from active synapses. We used a neuronal-synaptobrevin-spGFP_1-10_ chimera (nSyb-spGFP_1-10_) instead of CD4-spGFP_1-10_ (Fig. 5i). nSyb-spGFP_1-10_ is exposed only after presynaptic neuronal activation and, therefore, preferentially labels active synapses^67^. The freshly isolated live brain was exposed to KCl (three times, 5 s each time) to induce depolarization^67^. When DTk^5Fa^-Gal4 drove the expression of nSyb-spGFP_1-10_ and R18H11-LexA drove the expression of spGFP11, nSyb-GRASP signal was produced (Fig. 5i, −). Application of KCl enlarged this nSyb-GRASP signal (Fig. 5i, + and Fig. 5k, +). When reciprocal nSyb-GRASP partners were used, no GFP signal appeared (Fig. 5j). These results further indicated that the DTk neurons were presynaptically innervated the DN1_p_s. Indeed, DTk^5Fa^-Gal4-driven CD8::GFP reporter revealed that dCLK labeled DN1_p_s contacted by the neurites of DTk neurons (Fig. 5l).

**Fig. 6:**
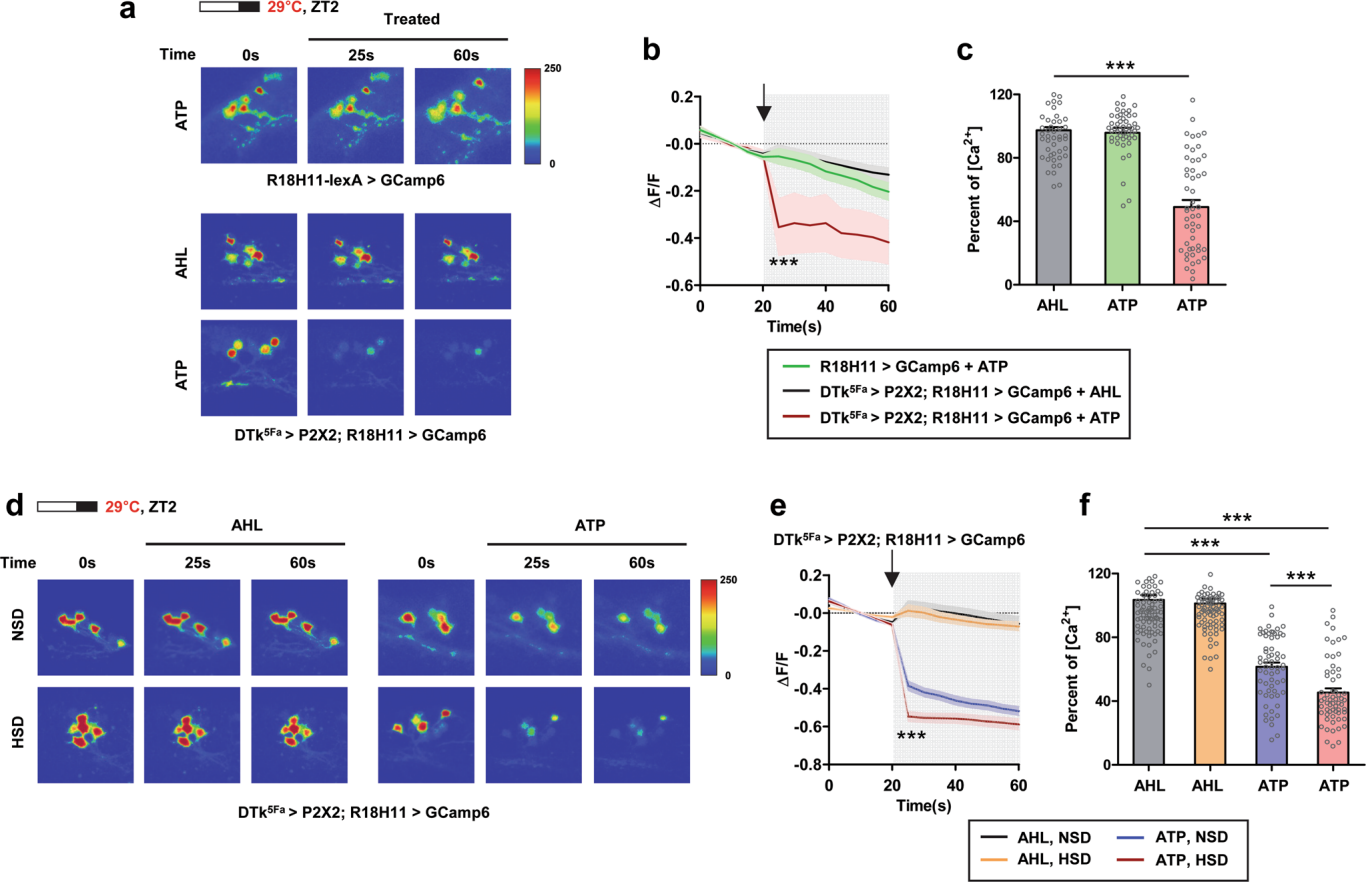
DTk neuron reduced intracellular Ca^2+^ levels in DN1_p_s. **a**–**f** Flies of the indicated genotypes were maintained on a 16L:8D cycle at 29 °C. On day 7, brains were dissected at ZT2 ~ 4. **a**, **d** Images with GCaMP-positive DN1_p_s following application of AHL or ATP. **b**, **e** ΔF/F values over time following AHL or ATP application (arrow) are shown. **c**, **f** Relative fold changes of intracellular Ca^2+^ levels. **c** ΔF/F values were normalized to AHL applied DTk^5Fa^ > P2X2; R18H11 > GCamp6 flies at 25 s. Bars indicate mean ± SEM (*n* = 45–51). Statistically significant differences between AHL and ATP treated groups (independent *t* test): ***P < 0.001. **f** ΔF/F values were normalized to AHL applied DTk^5Fa^ > P2X2; R18H11 > GCamp6 flies fed with a NSD at 25 s. Bars indicate mean ± SEM (*n* = 61–89). Statistically significant differences between AHL and ATP treated groups or between NSD and HSD condition (independent *t* test): ***P < 0.001.

To further examine the functionality of this connection, we expressed P2X2, a mammalian ATP receptor, in DTk^5Fa^-Gal4 cells, and GCaMP6, a fluorescent Ca^2+^ sensor, in R18H11-DN1_p_s. While R18H11 cells did not respond to ATP addition, R18H11 cells with DTk^5Fa^–Gal4 driving P2X2 expression showed a 60% decrease in Ca^2+^ levels after the addition of ATP compared to the AHL treated controls (Fig. 6a–c). These results indicated an inhibitory connection between in DTk^5Fa^ and R18H11-DN1_p_s. We next examined whether diets affected Ca^2+^ response. Consistent with the increase in synaptic contacts in flies fed a HSD, these flies showed a much greater decrease in intracellular Ca^2+^ levels in R18H11-DN1_p_s compared to the flies fed a NSD (Fig. 6d–f). We also noted the ATP response was observed in all GCaMP6-positive cells, indicating that DTk neurons innervated most if not all R18H11-DN1_p_s. This structural and physiological plasticity of the DTk and DN1_p_ circuit in flies fed a HSD was not unique to the high-temperature and long-photoperiod condition and were observed in flies maintained under the standard 12L:12D cycle at 25 °C conditions (Supplementary Fig. 8). Collectively, our results imply that the suppression of R18H11-DN1_p_s by DTk neurons extended M activity. Our results are consistent with a previous report that the optogenetic inhibition of R18H11 from the midday extended the E activity, but the timing is different^68^. In addition, the thermogenetic activation of R18H11-DN1_p_s promoted activity around dawn followed by siesta^29,68^. Thus, we think that DTk neurons regulate R18H11-DN1_p_s activity in a temporally gated manner in a way to suppress sleep-promoting output from DN1_p_s.

### Subsets of DN1_p_s were TkR86C-positive and required for M activity extension in HSD

**Fig. 7:**
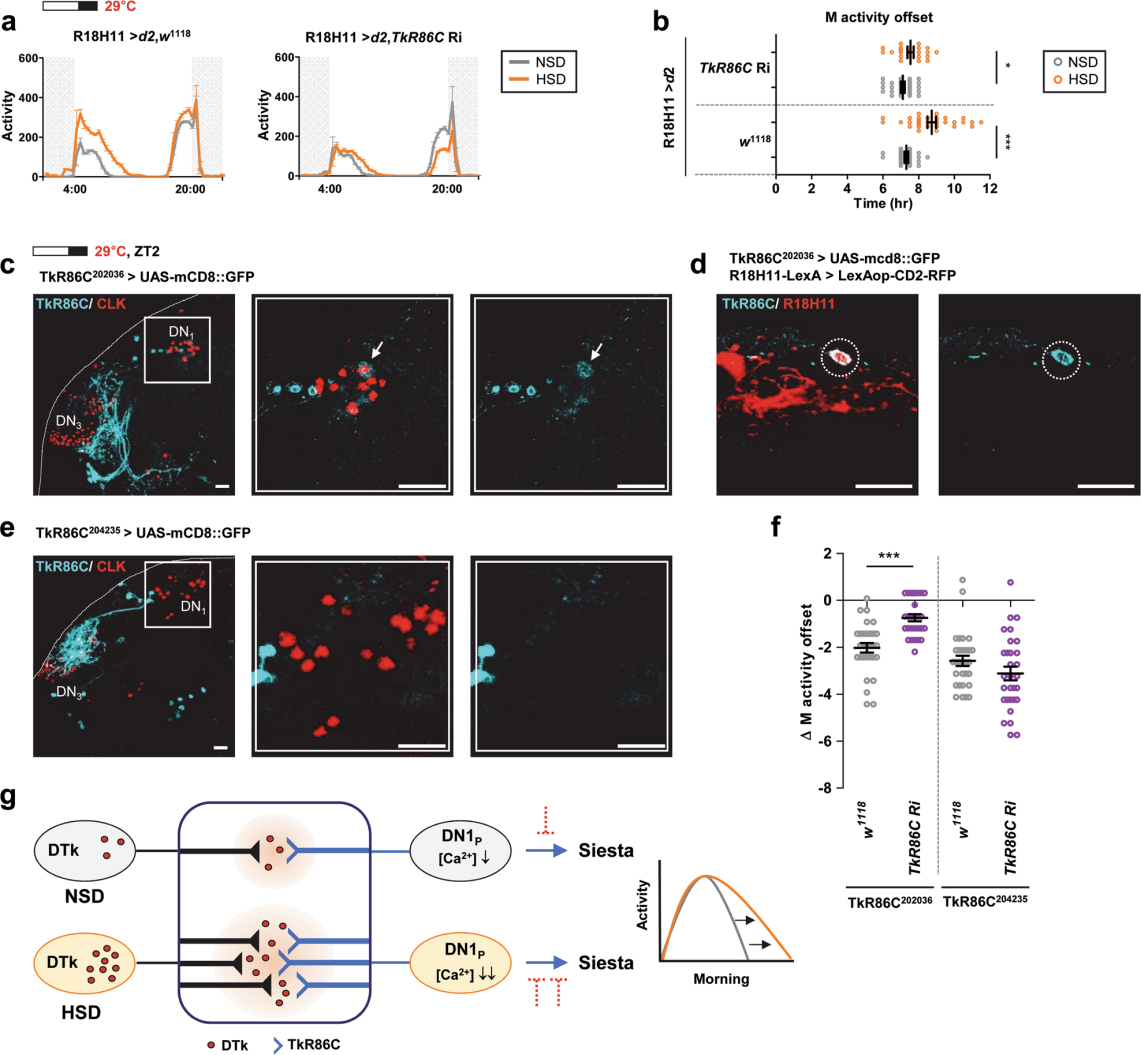
Subsets of DN1_p_s were TkR86C-positive and were required for M activity extension in HSD. **a**, **b** Locomotor activities of given genotypes of flies (denoted on top) were analyzed in NSD and HSD on a 16L:8D cycle at 29 °C. Daily activity profiles of flies on day 7 are shown. **b** M activity offset of individual flies on day 7 is shown. Bars indicate mean ± SEM values (*n* = 22–30). Statistically significant differences in M activity offset between control (R18H11 > *d2*, *w*^1118^) and *TkR86C* knockdown flies (R18H11 > *d*2, *TkR86C* Ri) (independent *t* test): *P < 0.05, ***P < 0.001. **c**, **e** Flies of the indicated genotypes (denoted on top) were maintained on a 16L:8D cycle at 29 °C. Brains were dissected at ZT2 and stained with anti-GFP (cyan blue) and anti-CLK (red) antibodies. The middle and right panels show a magnified image of the boxed region in the left panel. Arrow indicates a TkR86C-positive DN1_p_. All scale bars represented 20 μm. **d** Brains were stained with anti-GFP (cyan blue) and anti-RFP (red) antibodies. Dashed circle marks R18H11 and TkR86C positive cells in DN1_p_ region. All scale bars represented 20 μm. **f** Differences in M activity offset on day 7 between NSD and HSD groups (ΔM activity offset) for given genotypes of flies are shown (*n* = 26–31). Statistically significant differences in ΔM activity offset between control (TkR86C^202036^ > *w*^1118^ or TkR86C^204235^ > *w*^1118^) and *TkR86C* knockdown flies (TkR86C^202036^ > *TkR86C* Ri or TkR86C^204235^ > *TkR86C* Ri) (independent *t* test): ***P < 0.001. **g** Schematic of our model for a HSD-induced M activity extension in flies. *DTk* signaling is transmitted via TkR86C receptors onto postsynaptic DN1_p_s. The activation of DTk neurons reduced intracellular Ca^2+^ levels in DN1_p_s indicating the inhibitory connection between two neurons. A HSD increased the connections between DTk and DN1_p_s anatomically and physiologically. DN1_p_s promote activity at dawn and sleep at midday, we hypothesized that *DTk* modulates DN1_p_s activity in a time-gated manner to inhibit siesta, leading to the M activity extension (marked as a dashed line, because it was not proven in our study).

To further demonstrate that DN1_p_s innervated by DTk neurons mediated the M activity extension in HSD, we knocked down *TkR86C* using R18H11-Gal4. Compared with the control flies, the R18H11-Gal4-driven *TkR86C* knockdown abolished the effect of a HSD on M activity (Fig. 7a, b). To determine whether DN1_p_s expressed TkR86C, TkR86C-expressing cells were marked by TkR86C-Gal4-driven mCD8::GFP reporter and DN1_p_s were visualized using dCLK immunostaining. A single DN1_p_ pair was positive for both TkR86C^202036^-Gal4 (Fig. 7c) and R18H11-LexA (Fig. 7d). Another TkR86C-Gal4 line, TkR86C^204235^-Gal4, expressed the mCD8::GFP reporter similarly to TkR86C^202036^-Gal4 in the lateral dorsal brain region, but no DN1_p_s were positive (Fig. 7e). When TkR86C expression was downregulated using TkR86C^202036^-Gal4, the M activity offset delay in flies fed a HSD was mitigated compared with the control, but not completely suppressed (Fig. 7f and Supplementary Fig. 9a). Since the DTk neuron-dependent Ca^2+^ signal was observed in most of R18H11-DN1_p_s (Fig. 6), it seems likely that TkR86C^202036^-Gal4 did not target all the TkR86C-positive DN1_p_s. Nevertheless, TkR86C^204235^-Gal4-driven downregulation of TkR86C did not affect the HSD-induced M activity offset delay (Fig. 7f and Supplementary Fig. 9b).

Taken all together, this study revealed that *DTk* signaling onto DN1_p_s via *TkR86C* was required for HSD-induced M activity extension. A HSD augmented the inhibitory synaptic connection between DTk neuron and R18H11-DN1_p_s, likely suppressing the siesta-promoting activity of DN1_p_s (Fig. 7g).

## Discussion

In this study, we found that *D. melanogaster* had extended the M activity without much effect on E activity in a high-nutrient condition. *DTk* signaling onto DN1_p_s via *TkR86C* mediated this behavior with a concomitant increase in anatomical and physiological synaptic contacts between DTk neurons and DN1_p_s. It is known that DN1_p_s integrates environmental stimuli such as light and temperature for daily locomotor activity and sleep regulation^19–23^, and our results further indicated that DN1_p_s also coordinated the metabolic input via *DTk* signaling which shaped daily locomotor behavior.

*Tk* constitutes an evolutionarily well-conserved family of brain/gut neuropeptides that function as important neuromodulators in the central and peripheral nervous systems (reviewed in ref. ^51^). The mammalian Tk family members are substance P (SP), neurokinin A, and neurokinin B, which are produced from the pre-protachykinin-A gene. SP plays important modulatory roles in many processes (e.g., sensory processing, pain transmission, neurogenic inflammation, and stress) and has been implicated in the regulation of the circadian timing system. In the photic entrainment pathway, glutamatergic signals following photoreception are transmitted to the suprachiasmatic nucleus master clock of the mammalian circadian timing system. This process is enhanced by SP via the NK1 receptor (NK1R)^69–71^. Interestingly, decreasing NK1R by an antagonist attenuates the light pulse-induced phase shift during the late-night but not during the early night^72^. SP enhances acetylcholine release in the limbic/prefrontal area during the morning, but not during the afternoon^73^. These findings suggest that SP affects the circadian timing system during a time-restricted window, which is consistent with our results showing *DTk*-associated morning-restricted effects on locomotor behavior in flies on a HSD. In addition, our study suggested that SP might also be implicated to signal metabolic input in mammals.

*DTk* and two TkRs, *TkR86C* and *TkR99D*, are homologs of SP and its receptor NK1R, respectively^46,47^. Similar to mammalian Tk, the *DTk* gene encodes a pre-protachykinin that is processed into Tk-1–6^57,74^. In the CNS, *DTk* sensitizes sensory processing^52^ and participates in regulating systemic responses, including locomotor activity^52,53^, metabolic stress resistance^75^, and aggressive behavior^50^. While pan-neuronal or pontine neuronal knockdown of *DTk* increases activity or rest-activity bouts, respectively^52,53^, we did not observe an obvious change in locomotor activity in *DTk* knockdown flies fed a NSD (Fig. 3c). It appears that the effect of *DTk* on the regulation of general locomotor activity is not substantial. However, we found that *DTk* controlled the daily locomotor activity profile depending on the flies’ nutritional status, providing a novel neuromodulatory role for *DTk* in the CNS. A previous report that DTk expressed in five pairs of large protocerebral neurosecretory cells (designated ipc-1 and ipc-2a) regulates metabolic stress responses further supports the role of *DTk* in the regulation of metabolism in the CNS^75^. We found that in flies fed a HSD, there were increased levels of intracellular *DTk* in the brain. In the midgut, starvation promotes intracellular *DTk* production, but only amino acids, not sucrose or coconut oil, affect *DTk*^76^, suggesting that the metabolic stimuli that induce *DTk* production might be different in the CNS versus the peripheral nervous system.

In this study, we found that DN1_p_s coordinated the metabolic input via *DTk* signaling and extend M activity. How DN1_p_s control this behavior? The activation of R18H11-DN1_p_s promotes activity around dawn^29^ but promotes midday sleep^68^. Neural circuits from DN1_p_s to drive activity and sleep have been identified. DN1_p_s promoting wakefulness project to the dorsomedial protocerebrum, pars intercerebralis (PI) region^19,29,30^. DN1_p_s also targets the ellipsoid body (EB) region via a subgroup of tubercular-bulbar (TuBu) neurons in the anterior region. This circuit appeared to be sleep promoting in one study^77^ and wake promoting in the other study^78^. Our internal Ca^2+^ measurements following DTk neuron activation showed that connections between DTk neurons and R18H11-DN1_p_s are inhibitory. Given that the enhancement of *DTk* signaling onto DN1_p_s extended M activity without affecting M activity onset, we hypothesized that *DTk* suppresses the siesta-promoting DN1_p_ circuit thereby extend the M activity in the temporally gated manner (Fig. 7g). Interestingly, optogenetic inhibition of R18H11 from the midday extended the E activity and that is consistent with our idea, yet the timing is different^68^. High nutrition impacted the flies’ locomotor activity largely in the morning. Our immunostaining data showed that DTk levels were higher in the morning in some DTk-expressing nuclei in the brain such as LPP1, LPP2, or SMP (Fig. 4c). The rhythmic oscillation of DTk in the specific nucleus mediating the HSD effect might be the underlying mechanism for DTk-associated morning-restricted HSD effects on locomotor behavior. The rhythmic presentation of TkR86C on DN1_p_s might cause phase-specific effects as well, which require further study. Given DN1_p_s excitability is maximal in the morning^79^, the inhibitory inputs from DTk neurons might have the strongest impact on DN1_p_s in the morning.

The effect of a HSD on M activity increased in an environment of a summer-like high temperature and a long photoperiod (Supplementary Fig. 1 and Fig. 2); however, the effect of HSD on structural and physiological connections between DTk5Fa neurons and R18H11-DN1_p_s were similar in flies reared in either 25 °C 12L:12D or 29 °C 16L:8D. These results suggested that temperature and photoperiod might have additive effects with the HSD on M activity in flies. Intriguingly, in vivo Ca^2+^ imaging indicates that DN1_p_s are inhibited by heating^80^ supporting our hypothesis of the additive effect of high temperature and *DTk*-mediated signaling both decreasing internal Ca^2+^. There is a previous report that temperature elevation to high levels (>30 °C) prolong morning activity and delay midday sleep onset, which is similar to the effect of a HSD in our study except prolonged morning activity is observed only in male flies^78^. The temperature information that affected sleep was transmitted to DN1_p_s also via two neuronal groups expressing TrpA1 (i.e., TrpA1[SH]-Gal4- and ppk-Gal4-active cells). The separate circuits appear to converge onto DN1_p_s to deliver temperature and high-nutrient information. The morning activity of the fly comprises the lights-on startle component, which is the sharp increase in activity and an endogenous circadian component, the morning peak^43^. Under the standard 12L:12D cycle, the two components are not separable because the circadian component is largely masked by the startle response. With a long photoperiod, circadian activity appears separately after the startle response. Therefore, we think that photoperiodic gating of circadian M activity is timely followed by *DTk* signaling, leading to enhancement of the M activity extension in long photoperiod.

Tachykinin receptors are G-protein-coupled receptors, and NK1R is usually coupled to the Gq/11 cascade, leading to an increase in internal Ca^2+^ (reviewed in ref. ^81^). However, interaction with other G proteins and diverse downstream signaling pathways had been also known in different tissues (reviewed in ref. ^82^). In Drosophila, *DTk* also increases Ca^2+^ levels in *TkR99D*-transfected HEK293 cells suggesting an increase in neural activity^45,83^. On the other hand, olfactory receptor neurons expressing *TkR99D* are suppressed by *DTk*, suggesting that *DTk* mediates inhibitory neuromodulation^84,85^. While the TkR86C downstream intracellular signaling pathway in *Drosophila* is unknown, in this study we showed that DTk^5Fa^ neuron activation reduced intracellular Ca^2+^ levels of R18H11-DN1_p_s possibly via TkR86C (Figs. 6 and  7).

Mice fed a HFD exhibit reduced rhythmicity and lengthened periods of activity^10^ with slower responses to light^86^; however, whether a HFD affects the locomotor activity profile of mice as it does in flies is unknown. A hypocaloric diet with restricted feeding advances mouse activity onset without a change of period^87^, which is comparable to the delayed M activity offset of flies in HSD in the opposite direction^87^. The question remains, why did flies in a high-nutrient diet show extended locomotor activity only in the morning? The total activity was generally higher in flies fed a HSD than a NSD, but the increase for the *w*^1118^ flies was not significant. Thus, it is conceivable that flies may increase their locomotor activity to balance energy input and expenditure but may restrict this change to the morning phase not to compromise a deep sleep during the night phase^88,89^.

## Methods

### Fly stocks

*TkR86C*^▵F28^ flies^50^ were provided by David Anderson (California Institute of Technology, USA). UAS-mCD8::GFP;lexAop-CD2 RFP flies were provided by Seok Jun Moon (Yonsei University, Republic of Korea). UAS-CD4-spGFP_1-10_;lexAop-CD4-spGFP_11_ (BL58755), UAS-nSyb-spGFP_1-10_;lexAop-CD4-spGFP_11_ (BL64314), and lexAop-nSyb-spGFP_1-10_;UAS-CD4-spGFP_11_ (BL64315) flies were provided by Chunghun Lim (UNIST, Republic of Korea). The pdf-Gal4^90^ flies were a gift from Jae H. Park (University of Tennessee, USA). The following lines were obtained from the Bloomington Drosophila Stock Center: *w*^1118^(BL5905), elav-Gal4^C155^ (BL458), elav-GS-Gal4 (BL43642), DTk^2Ma^-Gal4 (BL51973), DTk^5Fa^-Gal4 (BL51975), R65E09-Gal4 (BL39358), UAS-*TkR99D* RNAi (BL55732), UAS-GFP.nls (BL4776), 10XUAS-IVS-mCD8::GFP (BL32187), UAS-dTrpA1(BL26263), R18H11-Gal4 (BL48832), R18H11-lexA (BL52535), pdf-lexA (BL52685), UAS-P2X2 (BL91222), 13XLexAop2-IVS-GCaMP6m (BL44276). The following fly stocks were obtained from the Vienna Drosophila Resource Center: UAS-*DTk* RNAi (V103662), UAS-*TkR86C* RNAi (V13392), TkR86C^039622^-Gal4 (V202036), TkR86C^039625^-Gal4 (V204235). Neuropeptide RNAi lines: DTk(V103662), Dh44(V108473), Ptth(V102043), FMRFa(V103981), Nplp1^1^(V107116), Capa(V101705), Burs(V102204), Mip(V106076), Nplp1^2^(V14035), NPF(V108772), Proc(V102488), Nplp3(V105584), Akh(V105063), AstA(V103215), Dh31(V50295), Nplp4(V104662), Ms(V108760), Pdf(BL25802), Dsk(V14201), AstC(V102735), Ilp3(V106512), Ilp5(V105004), Acp26Aa(V41193), Pburs(V102690), Ilp2(V44761), Hug(V107771), hec(V7223), BomS2(V10586), Lgr1^1^(V104877), ETH(V18825), Lk(V14091), natalisin(V19547), Lgr1^2^(V13566), ITP(V43848). elav-Gal4, elav-GS-Gal4, and pdf-Gal4 were crossed to UAS-dicer2/CyO to generate dcr2;elav-Gal4, dcr2;elav-GS-Gal4, and dcr2;pdf-Gal4 and used as driver flies for knockdown of expression. *w*^1118^ (BL5905) flies were used as a background strain in this study. UAS-*DTk* RNAi and UAS-*TkR86C* RNAi were outcrossed to *w*^1118^ (BL5905) for six generations.

### Locomotor behavior analysis

Locomotor activity of individual flies was determined using the Drosophila Activity Monitoring System 3 software (Trikinetics, version 1.02). Young male flies were used for the analysis and maintained in glass tubes containing 2% agar and 5% sucrose (normal sucrose diet, NSD) or 30% sucrose (high-sucrose diet, HSD) or 5% sucrose-containing 20% coconut oil (Nutiva) (high-fat diet, HFD). Flies were kept in incubators at the indicated temperature (25 °C or 29 °C) and were exposed to a 12L:12D or 16L:8D cycle for the indicated number of days of the experiment. Averaged fly locomotor activity profiles were plotted using GraphPad Prism5 software. To obtain the M and E activity phases, the onset/offset formula [(A_n+2_ + A_n+1_) – (A_n-1_ + A_n-2_) = ∆Activity] was used^91^. For M and E onset assessments, the largest 1-h increase in the activity window before light-on (M) or light-off (E) transitions, respectively, was used. For M and E offset assessments, the largest 1-h decrease in the activity window after light-on (M) or light-off (E) transitions, respectively, was used. Activities during the first 30 min after the light-on/off transition were removed to minimize the light-induced startle response.

### Food intake assays

To quantify the food intake of the flies, the absorbance of ingested dye was measured following method with slight modification^92^. Flies were maintained at 16L:8D cycle at 29 °C. Flies in groups of 16 were collected at ZT2 and starved for 18 h in 2% agar. Then flies were allowed to feed on 5% sucrose in 2% agarose for 20 min, transferred to new vials containing 1% blue dye (McCormick), and left to feed for another 15 min. Flies were homogenized in PBS, centrifuged for 3 min, and the absorbance of the blue dye in the supernatant was measured at 620 nm.

### Antibody production

We raised guinea pig anti-DTk antiserum (DTk-gp2) using the full-length protein as the antigen (Young In Frontier, Korea). We raised guinea pig anti-CLK antiserum (CLK-gp2) using the C-terminal 1138–3081 amino acids of the protein as the antigen (Young In Frontier, Korea). Antibody was purified from the antiserum with antigens immobilized on PVDF membranes. The antibody was dialyzed in PBS; glycerol (final 30%, v/v) was added as a stabilizer.

### Immunohistochemistry and confocal imaging

Immunostaining was performed as described previously with minor modifications^93^. Fly heads were cut open, fixed in 2% formaldehyde, and washed with 0.5% PAXD buffer (1× PBS, 5% BSA, 0.03% sodium deoxycholate, 0.03% Triton X-100)^94^. The fixed heads were dissected, and the isolated brains were permeabilized in 1% PBT for 20 min and then blocked in 0.5% PAXD containing 5% horse serum for 1 h. The following primary antibodies were diluted 1:200 and added directly to the mixtures: anti-DTk antibody (Gp2), anti-PDF antibody (C7) (DSHB), anti-PER antibody (Rb1)^95^, anti-TIM antibody (Rb1)^93^, anti-CLK antibody (Gp2), anti-GFP antibody (MBL International), anti-RFP antibody (MBL International), and anti-NC82 antibody (DSHB). The brains were washed with PAXD and incubated overnight with secondary antibodies in a blocking solution at 4 °C. The following secondary antibodies were used at a 1:200 dilution: goat anti-rabbit Alexa-488 (Thermo Fisher Scientific), goat anti-guinea pig Alexa-555 (Thermo Fisher Scientific), goat anti-mouse Alexa-555 (Thermo Fisher Scientific), and goat anti-mouse Alexa-633 (Thermo Fisher Scientific). Stained brain samples were washed with PAXD, incubated in 0.1 M phosphate buffer containing 50% glycerol for 30 min, and mounted using a mounting medium. Confocal images were obtained using an LSM 800 confocal microscope (Carl Zeiss) and were processed using Zen software (ZEN Digital Imaging for Light Microscopy, Carl Zeiss, version 3.1). For signal quantification, the pixel intensity of each cell was determined using ImageJ software. The intensity was the average of at least eight brains for each genotype.

### GFP reconstitution across synaptic partners analysis

GFP Reconstitution Across Synaptic Partners (GRASP) was performed to detect membrane contacts between flies expressing the CD4::spGFP_1-10_ fragment in one neuronal type and the CD4::spGFP_11_ fragment in the other neuronal type using the GAL4/UAS and LexA/lexAop systems, respectively^64,65^. pdf-LexA or R18H11-LexA drivers were used to express CD4::spGFP_11_ in LN_v_s or DN1_p_s, respectively. CD4::spGFP_1-10_ was expressed in DTk neurons using the DTk^5Fa^-Gal4 driver. A modified GRASP (i.e., nSyb-GRASP) analysis was performed to determine whether the contacts between two neuronal groups were active synapses^67^. In the nSyb-GRASP system, neuronal synaptobrevin fused to spGFP_1-10_ (nSyb::spGFP_1-10_) fragment is expressed in one neuronal type. spGFP_1-10_ is exposed to the extracellular space following neuronal activation because n-Syb is a component of the synaptic vesicle membrane. R18H11-LexA and DTk^5Fa^-Gal4 drivers were crossed with either UAS-nSyb-spGFP_1–10_, lexAop-CD4-spGFP_11_ or UAS-CD4-spGFP_11_, lexAop-nSyb-spGFP_1-10_. To apply KCl to evoke neuronal activation, flies were anesthetized on ice, and their brains were dissected in adult hemolymph (AHL, containing 108 mM NaCl, 5 mM KCl, 4 mM NaHCO_3_, 1 mM NaH_2_PO_4_, 15 mM sucrose, 5 mM HEPES, 8.2 mM MgCl_2_, 2 mM CaCl_2_, pH 7.4). Dissected brains were rinsed quickly (5 s) three times with 70 mM KCl in AHL, and then imaged in AHL 20 min after KCl application. Control flies were rinsed in AHL containing no additional KCl and imaged the same way. GRASP Area was determined using the ImageJ software. A GFP (positive GRASP) signal above background levels was selected by adjusting the color threshold and the area of the GFP signal was obtained from ImageJ software (version 1.53c).

### GCaMP imaging and analysis

Adult male flies were entrained for 7 days in incubators at the indicated temperature and light cycle. From ZT2 to ZT4, flies were dissected in adult hemolymph-like buffer (AHL, 108 mM NaCl, 8.2 mM MgCl_2_, 4 mM NaHCO_3_, 1 mM NaH_2_PO_4_, 2 mM CaCl_2_, 5 mM KCl, 5 mM HEPES, 80 mM sucrose)^96^. Dissected *Drosophila* brains were rapidly mounted on a cover glass and sprayed with 20 μl of AHL buffer to prevent the brain from drying out. After stabilizing the samples for 3 min in AHL buffer, confocal imaging was performed to determine the baseline Ca^2+^ levels. ATP, at a concentration of 2.5 mM, or AHL (control) were applied directly to the AHL buffer covering the brain, and imaging was performed. The Z stack images were taken (three layers) every 5 seconds to measure all the DN1_p_s. Image processing and measurement of fluorescence intensity were performed in ZEN (black edition) and ImageJ programs. A sum-intensity Z-projection of each time interval was measured after combining the images using the ZEN program (orthogonal projection). GCaMP-positive regions of interest (DN1_p_S cells) were manually drawn and mean intensities were measured at each time interval using the ImageJ program. The ratio changes were calculated using the following formula: ΔF/F = (Fn − F_0_)/F_0_, where Fn was the mean intensity of GCaMP-positive cells, F_0_ was the average baseline intensity. Brains with cells that had unstable baselines were not used.

### qRT-PCR

The total RNA was extracted from fly heads using QIAzol reagent (QIAGEN). The total RNA (1 μg) was reverse transcribed using an oligo(dT)20 primer (for mRNA) and PrimeScript RTase (TaKaRa). Quantitative, real-time PCR (qPCR) was performed using Rotor Gene 6000 (QIAGEN) with TB Green Premix Ex Taq (Tli RNaseH Plus, TaKaRa). The following primers were used: DTk forward, 5′-CGGTCAATTCCTTTGTGGG-3′; DTk reverse, 5′-ATTCGGAGAGAGCTGCAC-3′; TkR86C forward, 5′-GACCAAGCACTATTACAATGG-3′; TkR86C reverse, 5′-GCCATAGAAGTGGGATATCG-3′; TkR99D forward, 5′-GTGGAGAATGTGCGGAGTAAG-3′; and TkR99D reverse, 5′-CGGGTAGCAGGATGTGATTATG-3′. Noncycling mRNA encoding *cbp20* was used to normalize gene expression with the primers *cbp20* forward, 5′-GTATAAGAAGACGCCCTGC-3′; and *cbp20* reverse, 5′-TTCACAAATCTCATGGCCG-3′. The data were analyzed using Rotor Gene Q- Pure Detection software (version 2.2.3), and the relative mRNA levels were quantified using the 2^−∆∆Ct^ method in which ∆∆Ct = [(C_t_ target − C_t_ *cbp20*) of the experimental group] − [(C_t_ target − C_t_ *cbp20*) of control group].

### Statistics and reproducibility

GraphPad Prism5 software was used for the statistical analysis. All population assays were performed with the experimental and control genotypes in parallel and with more than *n* = 16 flies per genotype. All data represented multiple independent experiments. Nonparametric *t* test statistics were used unless otherwise indicated.

### Reporting summary

Further information on research design is available in the Nature Research Reporting Summary linked to this article.

## Supplementary information

Supplementary Information

Description of Additional Supplementary Files

Supplementary Data 1

Reporting Summary

## Data Availability

The source data underlying the graphs are shown as Supplementary Data 1. All other data supporting the findings of this study are available from the corresponding author upon reasonable request.
