# Supplementary material for: Metabolic control of daily locomotor activity mediated by tachykinin in Drosophila
Source: Commun Biol. 2021 Jun 7;4:693. doi: 10.1038/s42003-021-02219-6 (PMC8184744; doi:10.1038/s42003-021-02219-6)
Supplement: Supplementary file 1 — Supplementary Information [file 42003_2021_2219_MOESM1_ESM.pptx]

## Slide 1
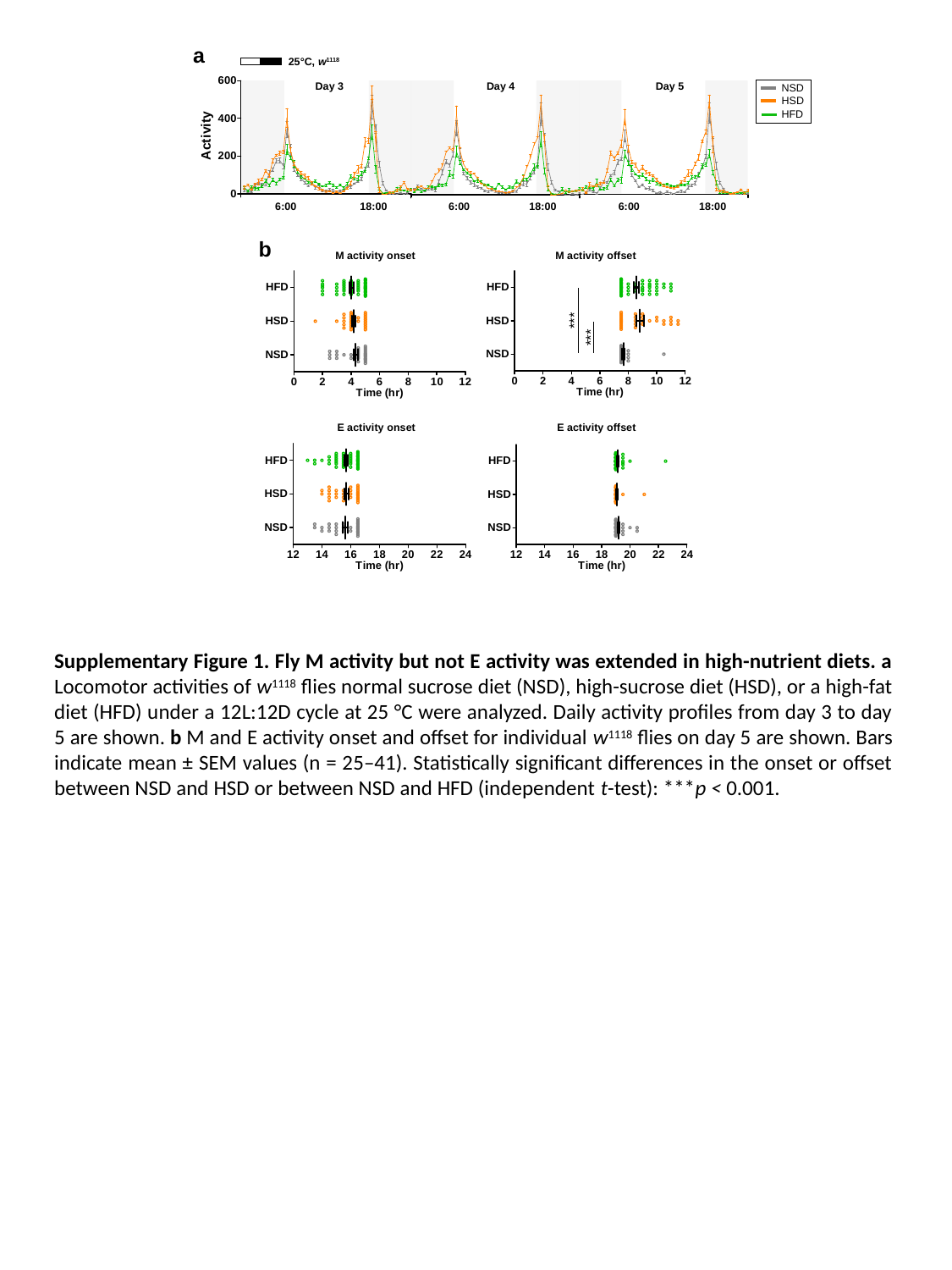

a
25°C, w1118
Day 3
Day 4
Day 5
NSD
HSD
HFD
6:00
18:00
6:00
18:00
6:00
18:00
b
M activity onset
M activity offset
***
***
E activity onset
E activity offset
Supplementary Figure 1. Fly M activity but not E activity was extended in high-nutrient diets. a Locomotor activities of w1118 flies normal sucrose diet (NSD), high-sucrose diet (HSD), or a high-fat diet (HFD) under a 12L:12D cycle at 25 °C were analyzed. Daily activity profiles from day 3 to day 5 are shown. b M and E activity onset and offset for individual w1118 flies on day 5 are shown. Bars indicate mean ± SEM values (n = 25–41). Statistically significant differences in the onset or offset between NSD and HSD or between NSD and HFD (independent t-test): ***p < 0.001.

## Slide 2
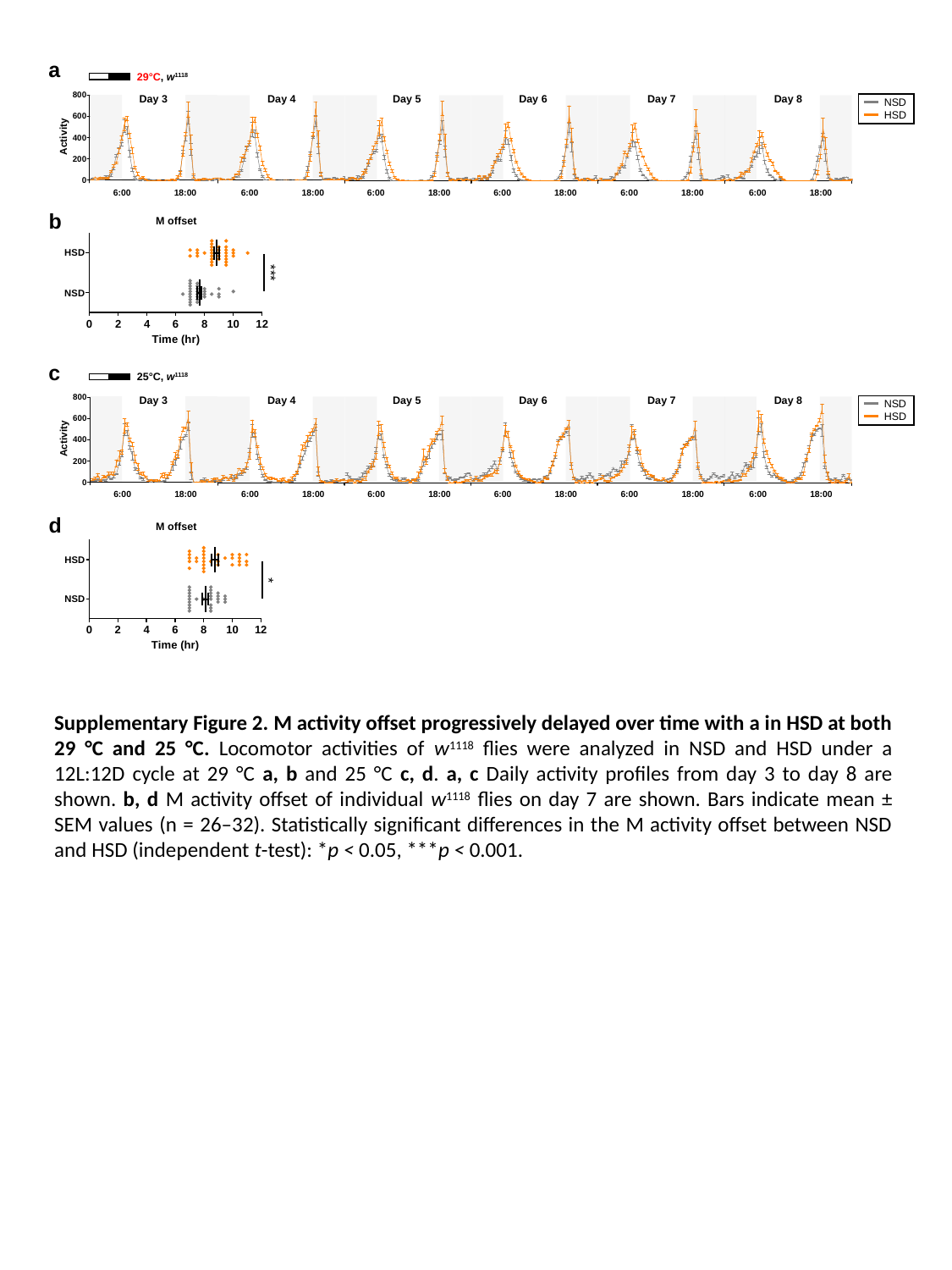

a
29°C, w1118
Day 6
Day 7
Day 8
Day 3
Day 4
Day 5
NSD
HSD
6:00
18:00
6:00
18:00
6:00
18:00
6:00
18:00
6:00
18:00
6:00
18:00
b
M offset
***
c
25°C, w1118
Day 6
Day 7
Day 8
Day 3
Day 4
Day 5
NSD
HSD
6:00
18:00
6:00
18:00
6:00
18:00
6:00
18:00
6:00
18:00
6:00
18:00
d
M offset
*
Supplementary Figure 2. M activity offset progressively delayed over time with a in HSD at both 29 °C and 25 °C. Locomotor activities of w1118 flies were analyzed in NSD and HSD under a 12L:12D cycle at 29 °C a, b and 25 °C c, d. a, c Daily activity profiles from day 3 to day 8 are shown. b, d M activity offset of individual w1118 flies on day 7 are shown. Bars indicate mean ± SEM values (n = 26–32). Statistically significant differences in the M activity offset between NSD and HSD (independent t-test): *p < 0.05, ***p < 0.001.

## Slide 3
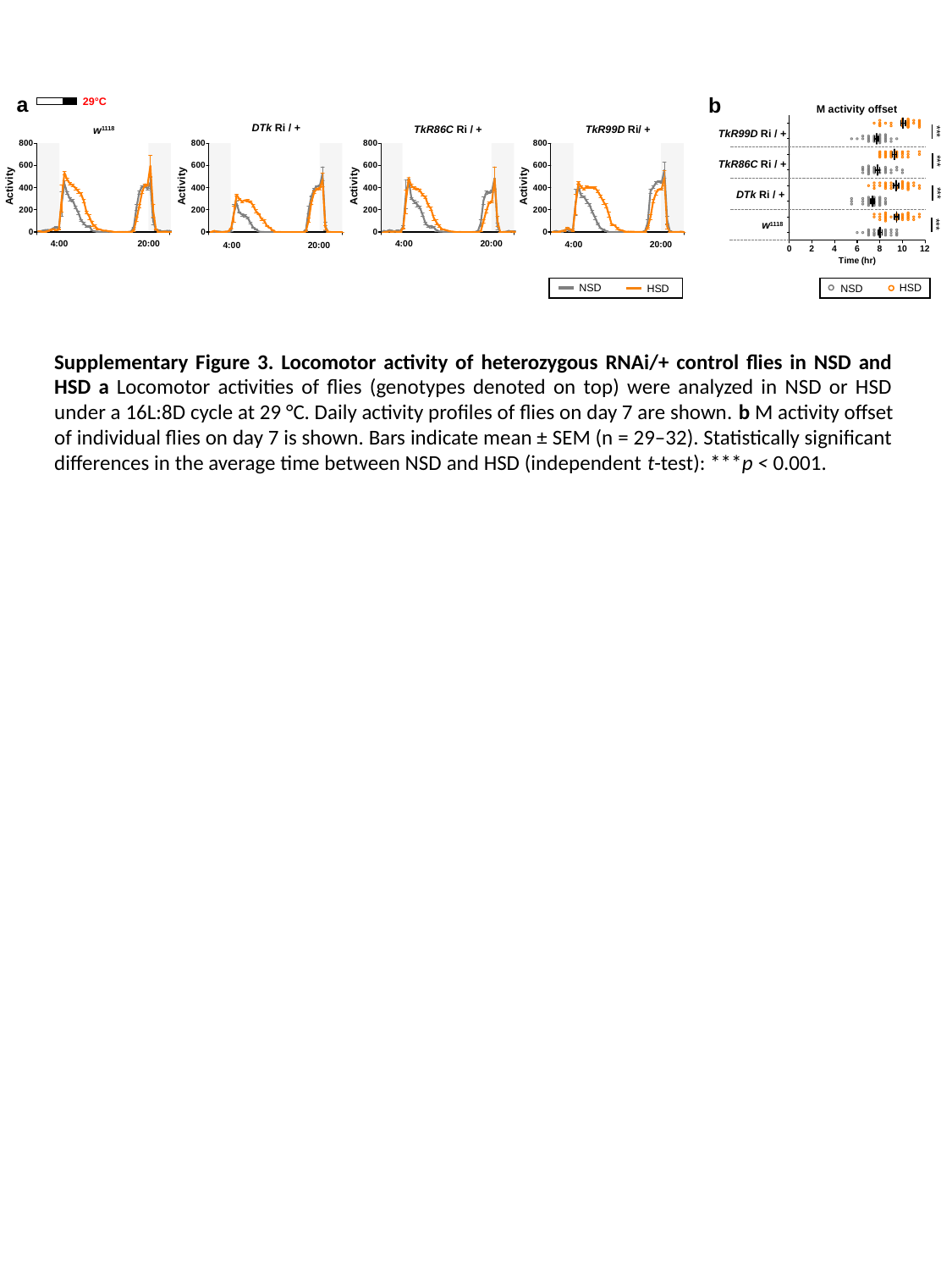

a
b
29°C
M activity offset
DTk Ri / +
TkR86C Ri / +
TkR99D Ri/ +
w1118
***
TkR99D Ri / +
***
TkR86C Ri / +
***
DTk Ri / +
***
w1118
4:00
20:00
4:00
20:00
4:00
20:00
4:00
20:00
NSD
HSD
HSD
NSD
Supplementary Figure 3. Locomotor activity of heterozygous RNAi/+ control flies in NSD and HSD a Locomotor activities of flies (genotypes denoted on top) were analyzed in NSD or HSD under a 16L:8D cycle at 29 °C. Daily activity profiles of flies on day 7 are shown. b M activity offset of individual flies on day 7 is shown. Bars indicate mean ± SEM (n = 29–32). Statistically significant differences in the average time between NSD and HSD (independent t-test): ***p < 0.001.

## Slide 4
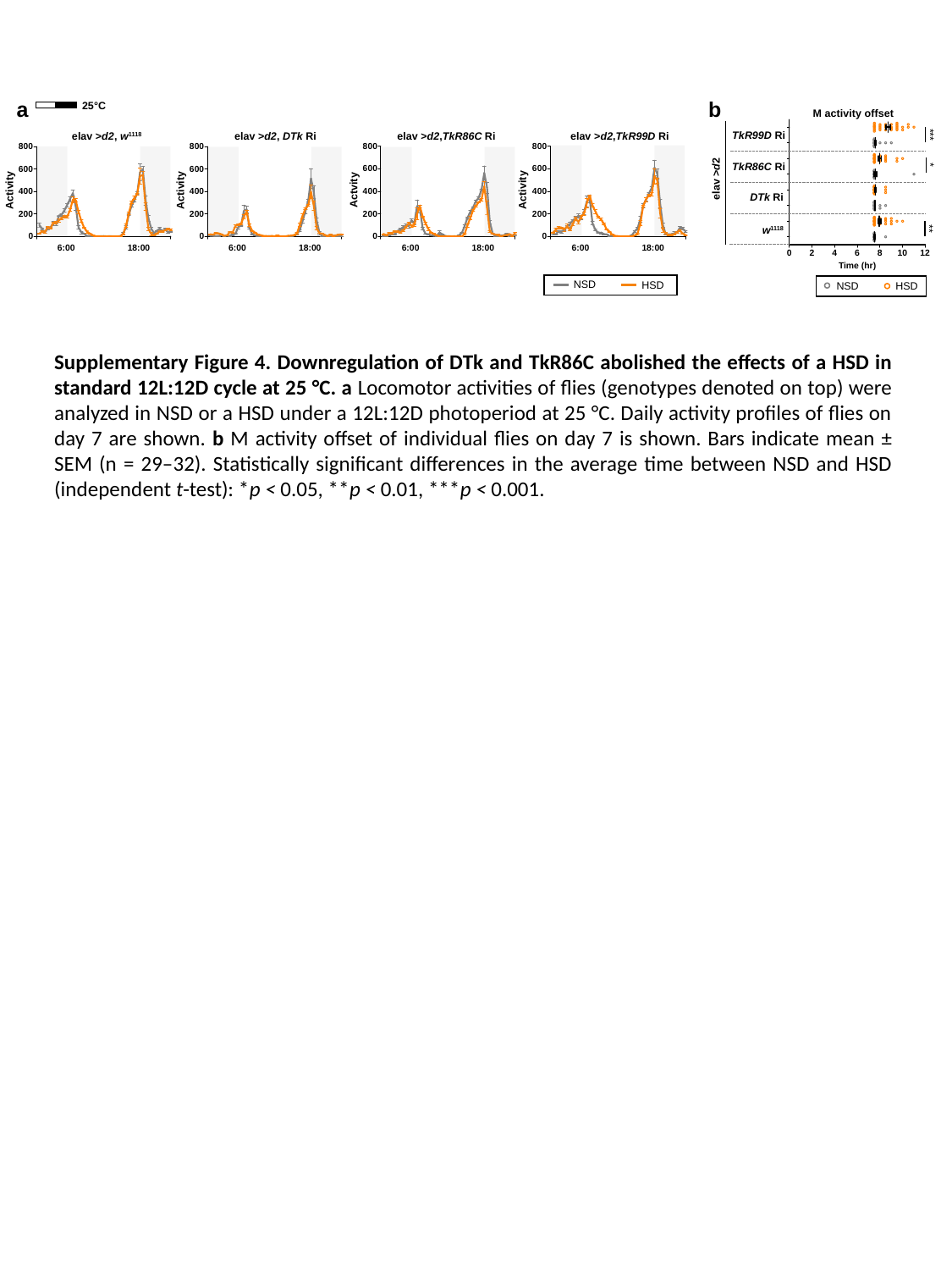

a
b
25°C
M activity offset
***
TkR99D Ri
elav >d2, w1118
elav >d2, DTk Ri
elav >d2,TkR86C Ri
elav >d2,TkR99D Ri
*
TkR86C Ri
elav >d2
DTk Ri
**
w1118
6:00
18:00
6:00
18:00
6:00
18:00
6:00
18:00
NSD
HSD
HSD
NSD
Supplementary Figure 4. Downregulation of DTk and TkR86C abolished the effects of a HSD in standard 12L:12D cycle at 25 °C. a Locomotor activities of flies (genotypes denoted on top) were analyzed in NSD or a HSD under a 12L:12D photoperiod at 25 °C. Daily activity profiles of flies on day 7 are shown. b M activity offset of individual flies on day 7 is shown. Bars indicate mean ± SEM (n = 29–32). Statistically significant differences in the average time between NSD and HSD (independent t-test): *p < 0.05, **p < 0.01, ***p < 0.001.

## Slide 5
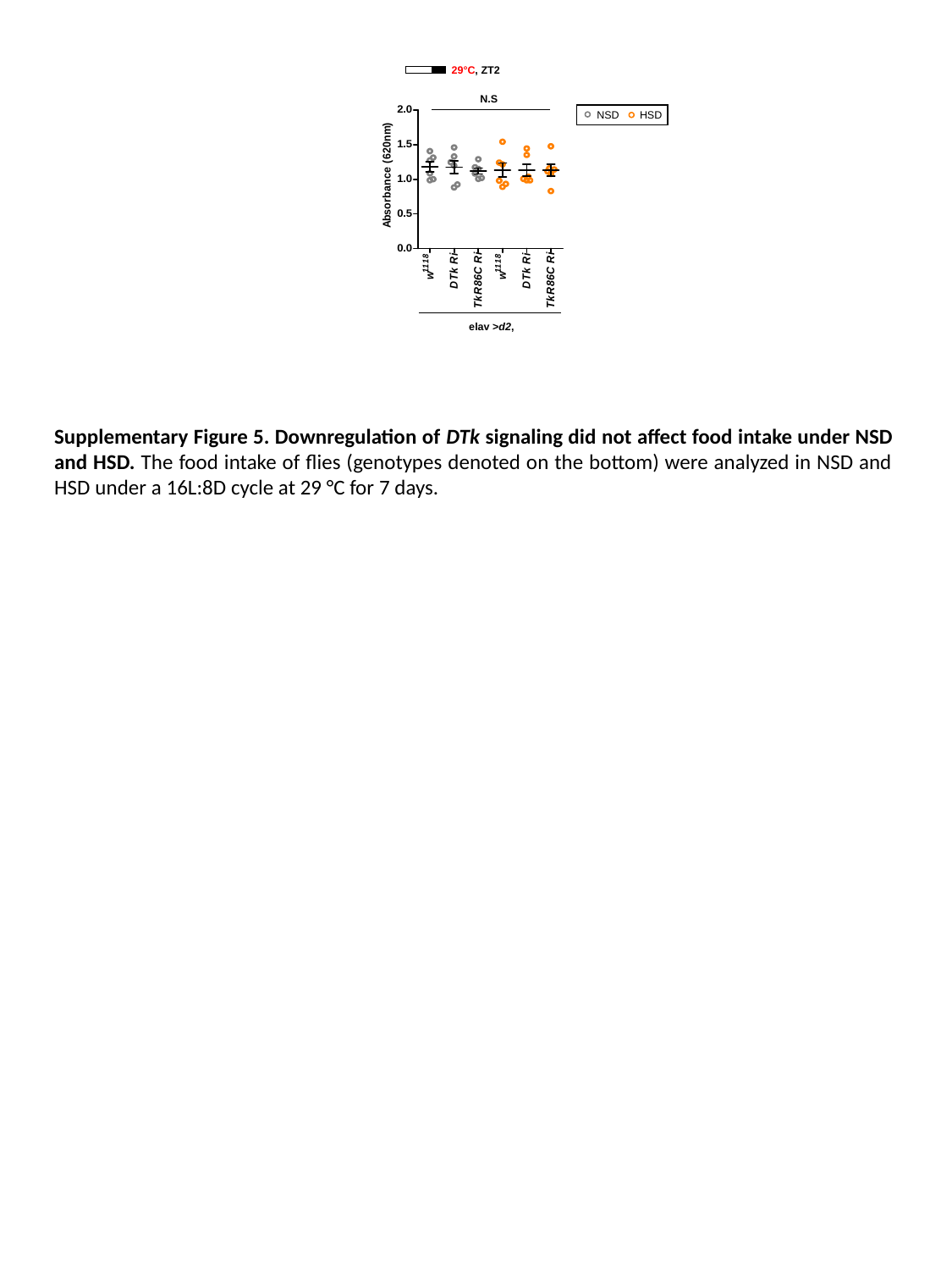

29°C, ZT2
N.S
HSD
NSD
elav >d2,
Supplementary Figure 5. Downregulation of DTk signaling did not affect food intake under NSD and HSD. The food intake of flies (genotypes denoted on the bottom) were analyzed in NSD and HSD under a 16L:8D cycle at 29 °C for 7 days.

## Slide 6
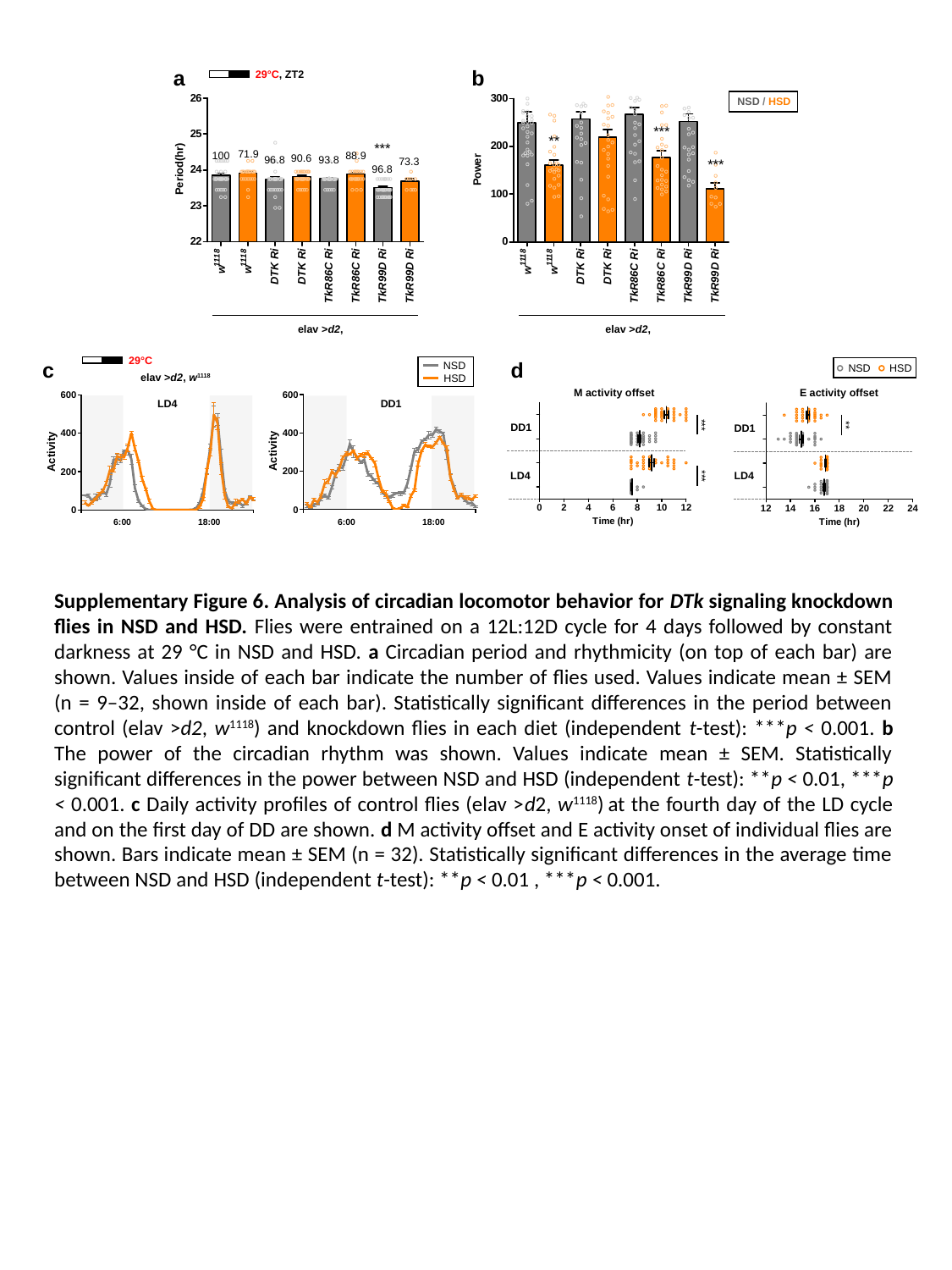

a
b
29°C, ZT2
NSD / HSD
***
**
***
71.9
88.9
100
90.6
96.8
93.8
73.3
***
96.8
32
23
30
29
29
24
30
9
elav >d2,
elav >d2,
29°C
c
d
NSD
HSD
NSD
HSD
elav >d2, w1118
E activity offset
M activity offset
LD4
DD1
***
**
DD1
DD1
LD4
LD4
***
6:00
18:00
6:00
18:00
Supplementary Figure 6. Analysis of circadian locomotor behavior for DTk signaling knockdown flies in NSD and HSD. Flies were entrained on a 12L:12D cycle for 4 days followed by constant darkness at 29 °C in NSD and HSD. a Circadian period and rhythmicity (on top of each bar) are shown. Values inside of each bar indicate the number of flies used. Values indicate mean ± SEM (n = 9–32, shown inside of each bar). Statistically significant differences in the period between control (elav >d2, w1118) and knockdown flies in each diet (independent t-test): ***p < 0.001. b The power of the circadian rhythm was shown. Values indicate mean ± SEM. Statistically significant differences in the power between NSD and HSD (independent t-test): **p < 0.01, ***p < 0.001. c Daily activity profiles of control flies (elav >d2, w1118) at the fourth day of the LD cycle and on the first day of DD are shown. d M activity offset and E activity onset of individual flies are shown. Bars indicate mean ± SEM (n = 32). Statistically significant differences in the average time between NSD and HSD (independent t-test): **p < 0.01 , ***p < 0.001.

## Slide 7
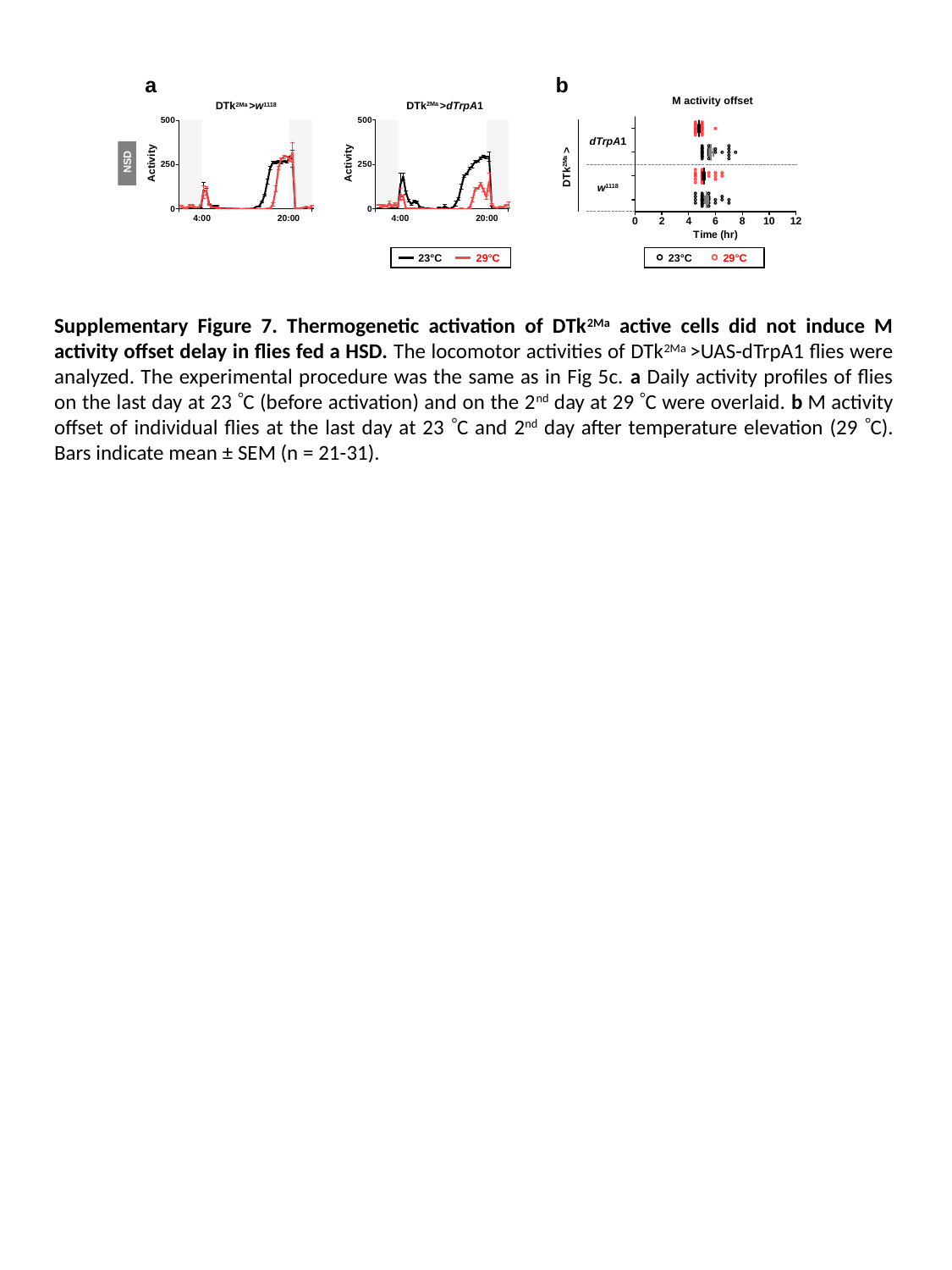

a
b
M activity offset
DTk2Ma >dTrpA1
DTk2Ma >w1118
dTrpA1
NSD
DTk2Ma >
w1118
4:00
20:00
4:00
20:00
23°C
29°C
23°C
29°C
Supplementary Figure 7. Thermogenetic activation of DTk2Ma active cells did not induce M activity offset delay in flies fed a HSD. The locomotor activities of DTk2Ma >UAS-dTrpA1 flies were analyzed. The experimental procedure was the same as in Fig 5c. a Daily activity profiles of flies on the last day at 23 °C (before activation) and on the 2nd day at 29 °C were overlaid. b M activity offset of individual flies at the last day at 23 °C and 2nd day after temperature elevation (29 °C). Bars indicate mean ± SEM (n = 21-31).

## Slide 8
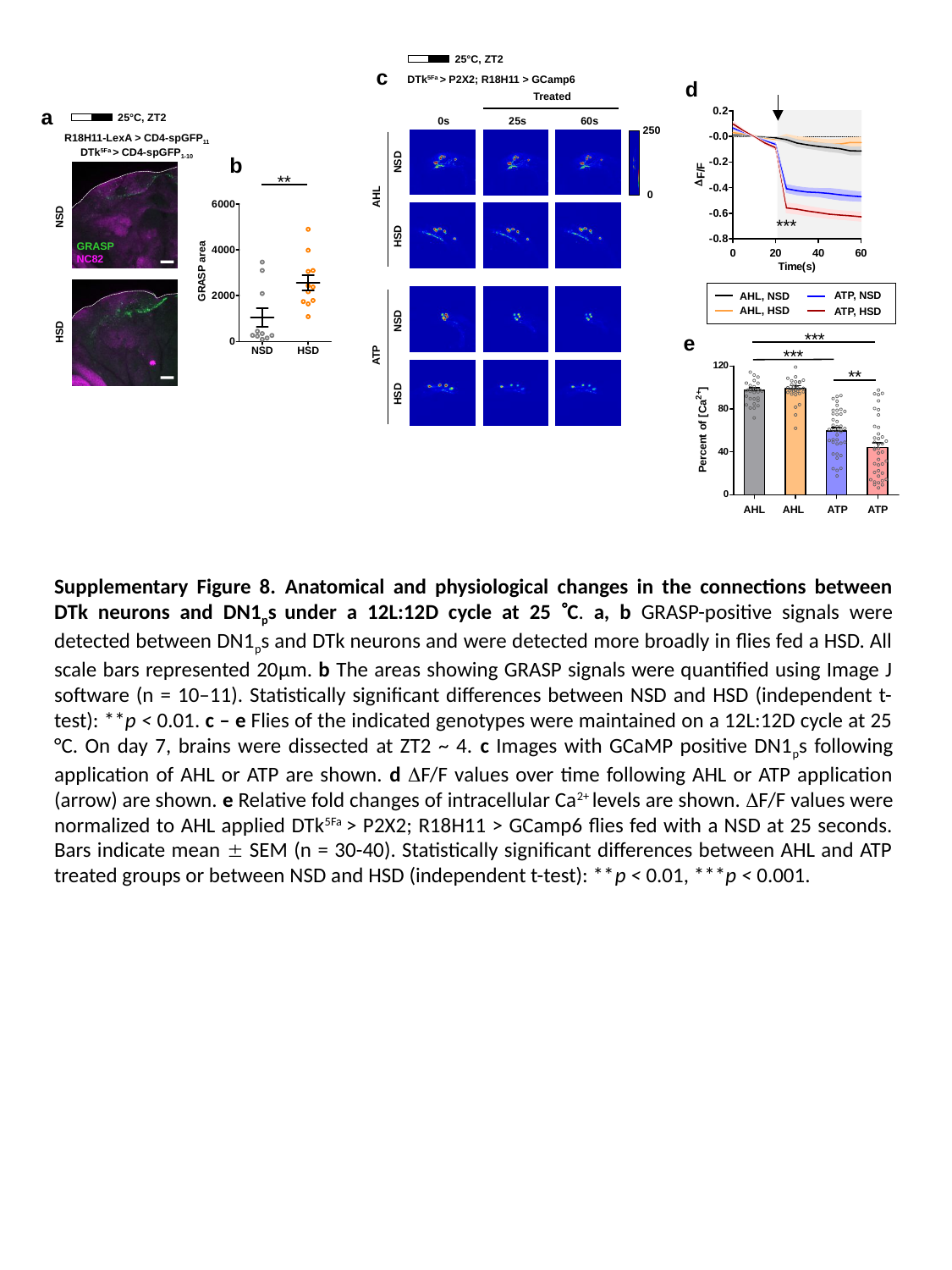

25°C, ZT2
c
DTk5Fa > P2X2; R18H11 > GCamp6
d
Treated
a
25°C, ZT2
0s
25s
60s
250
R18H11-LexA > CD4-spGFP11
DTk5Fa > CD4-spGFP1-10
b
NSD
**
0
AHL
NSD
***
HSD
GRASP
NC82
j
ATP, NSD
AHL, NSD
AHL, HSD
ATP, HSD
NSD
HSD
***
e
***
ATP
**
HSD
AHL AHL ATP ATP
Supplementary Figure 8. Anatomical and physiological changes in the connections between DTk neurons and DN1ps under a 12L:12D cycle at 25 °C. a, b GRASP-positive signals were detected between DN1ps and DTk neurons and were detected more broadly in flies fed a HSD. All scale bars represented 20μm. b The areas showing GRASP signals were quantified using Image J software (n = 10–11). Statistically significant differences between NSD and HSD (independent t-test): **p < 0.01. c – e Flies of the indicated genotypes were maintained on a 12L:12D cycle at 25 °C. On day 7, brains were dissected at ZT2 ~ 4. c Images with GCaMP positive DN1ps following application of AHL or ATP are shown. d ΔF/F values over time following AHL or ATP application (arrow) are shown. e Relative fold changes of intracellular Ca2+ levels are shown. ΔF/F values were normalized to AHL applied DTk5Fa > P2X2; R18H11 > GCamp6 flies fed with a NSD at 25 seconds. Bars indicate mean ± SEM (n = 30-40). Statistically significant differences between AHL and ATP treated groups or between NSD and HSD (independent t-test): **p < 0.01, ***p < 0.001.

## Slide 9
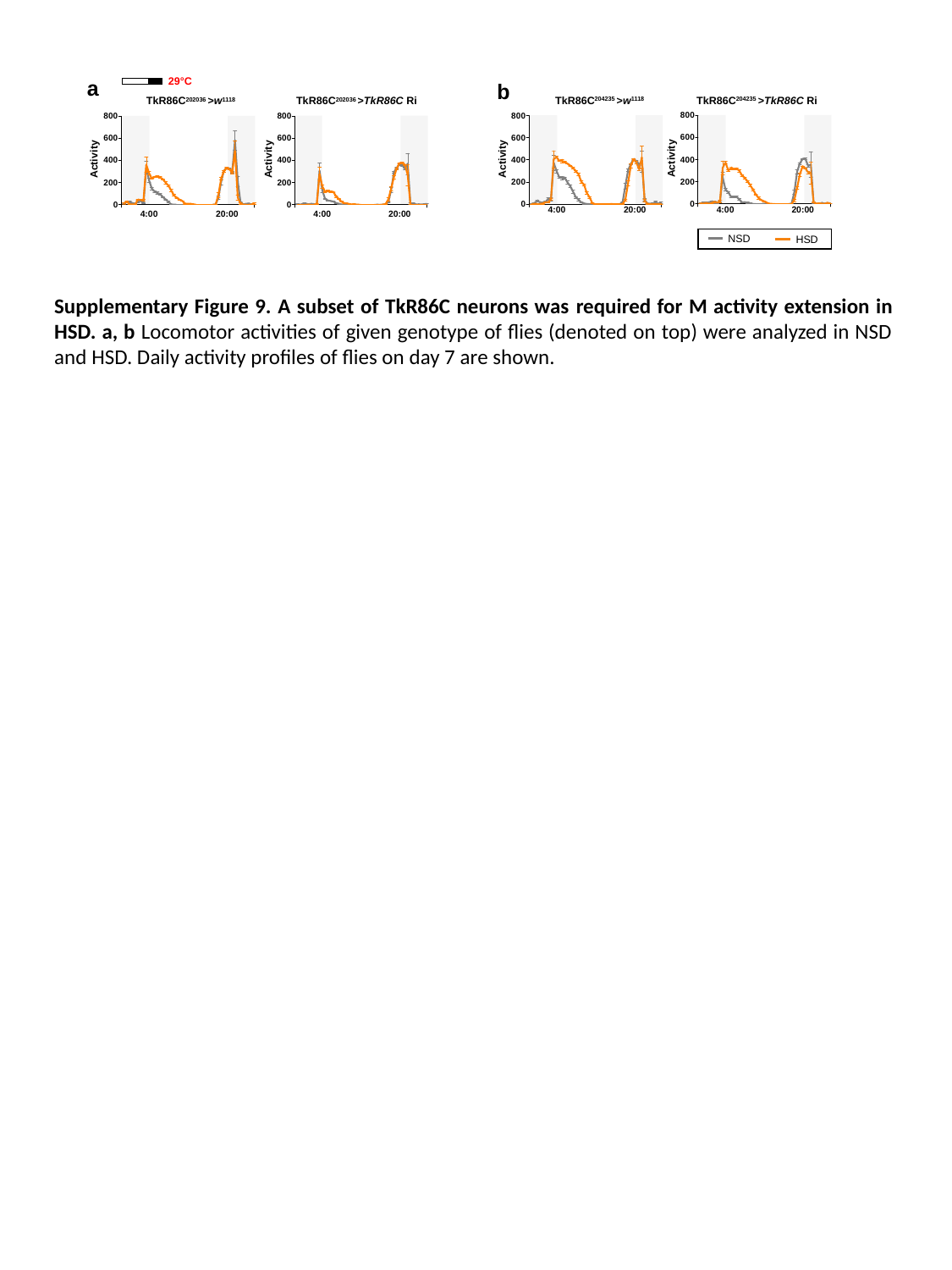

29°C
a
b
TkR86C204235 >w1118
TkR86C204235 >TkR86C Ri
TkR86C202036 >w1118
TkR86C202036 >TkR86C Ri
4:00
20:00
4:00
20:00
4:00
20:00
4:00
20:00
NSD
HSD
Supplementary Figure 9. A subset of TkR86C neurons was required for M activity extension in HSD. a, b Locomotor activities of given genotype of flies (denoted on top) were analyzed in NSD and HSD. Daily activity profiles of flies on day 7 are shown.
