# Supplementary material for: Metabolic control of daily locomotor activity mediated by tachykinin in Drosophila
Source: Commun Biol. 2021 Jun 7;4:693. doi: 10.1038/s42003-021-02219-6 (PMC8184744; doi:10.1038/s42003-021-02219-6)
Supplement: Supplementary file 2 — Description of Additional Supplementary Files [file 42003_2021_2219_MOESM2_ESM.pdf]

Description of Additional Supplementary Files

File name: Supplementary Data 1

Description: Source data underlying the graphs presented in the main figures and supplementary figures.
