# Supplementary material for: Metabolic control of daily locomotor activity mediated by tachykinin in Drosophila
Source: Commun Biol. 2021 Jun 7;4:693. doi: 10.1038/s42003-021-02219-6 (PMC8184744; doi:10.1038/s42003-021-02219-6)
Supplement: Supplementary file 4 — Reporting Summary [file 42003_2021_2219_MOESM4_ESM.pdf]

nature research

Corresponding author(s): Eun Young Kim

Last updated by author(s): May 12, 2021

Reporting Summary

Nature Research wishes to improve the reproducibility of the work that we publish. This form provides structure for consistency and transparency in reporting. For further information on Nature Research policies, see our Editorial Policies and the Editorial Policy Checklist.

Statistics

For all statistical analyses, confirm that the following items are present in the figure legend, table legend, main text, or Methods section.

n/a	Confirmed	
☐	☒	The exact sample size (*n*) for each experimental group/condition, given as a discrete number and unit of measurement
☐	☒	A statement on whether measurements were taken from distinct samples or whether the same sample was measured repeatedly
☐	☒	The statistical test(s) used AND whether they are one- or two-sided<br>*Only common tests should be described solely by name; describe more complex techniques in the Methods section.*
☒	☐	A description of all covariates tested
☐	☒	A description of any assumptions or corrections, such as tests of normality and adjustment for multiple comparisons
☐	☒	A full description of the statistical parameters including central tendency (e.g. means) or other basic estimates (e.g. regression coefficient) AND variation (e.g. standard deviation) or associated estimates of uncertainty (e.g. confidence intervals)
☐	☒	For null hypothesis testing, the test statistic (e.g. *F*, *t*, *r*) with confidence intervals, effect sizes, degrees of freedom and *P* value noted<br>*Give P values as exact values whenever suitable.*
☒	☐	For Bayesian analysis, information on the choice of priors and Markov chain Monte Carlo settings
☒	☐	For hierarchical and complex designs, identification of the appropriate level for tests and full reporting of outcomes
☒	☐	Estimates of effect sizes (e.g. Cohen's *d*, Pearson's *r*), indicating how they were calculated

Our web collection on statistics for biologists contains articles on many of the points above.

Software and code

Policy information about availability of computer code

Data collection	Confocal imaging data collection was performed using ZEN software from Carl Zeiss (version 3.1, blue edition)<br>Real-time quantitative RT-PCR data was collected using RRotor Gene Q- Pure Detection software from Qiagen (version 2.2.3)<br>Flies' locomotor activity data was collected using DAMsystem3 software from Trikinetics (version 1.02)
Data analysis	All graphing and associated statistical analysis was performed using GraphPad Prism 5 (version 5.01)<br>Confocal imaging data analysis was performed using ZEN software from Carl Zeiss (version 3.1, blue edition)<br>Confocal imaging signal quantification was performed using Image J software (version 1.53c)

For manuscripts utilizing custom algorithms or software that are central to the research but not yet described in published literature, software must be made available to editors and reviewers. We strongly encourage code deposition in a community repository (e.g. GitHub). See the Nature Research guidelines for submitting code & software for further information.

Data

Policy information about availability of data

All manuscripts must include a data availability statement. This statement should provide the following information, where applicable:

- Accession codes, unique identifiers, or web links for publicly available datasets
- A list of figures that have associated raw data
- A description of any restrictions on data availability

The source data underlying the graphs are shown as Supplementary data 1. All other data supporting the findings of this study are available from the corresponding author upon reasonable request.

Field-specific reporting

Please select the one below that is the best fit for your research. If you are not sure, read the appropriate sections before making your selection.

- [x] Life sciences
- [ ] Behavioural & social sciences
- [ ] Ecological, evolutionary & environmental sciences

For a reference copy of the document with all sections, see nature.com/documents/nr-reporting-summary-flat.pdf

Life sciences study design

All studies must disclose on these points even when the disclosure is negative.

Sample size	No statistical methods were used to predetermine sample size for experiments.
Data exclusions	Flies showing no activity count for 2 days were considered dead and data from those flies were excluded.
Replication	All the experiments shown were reliably reproducible.
Randomization	Because the samples are genetically modified flies and corresponding control flies, no randomization was performed.
Blinding	No blinding was performed. Blinding was unnecessary because the analysis is quantitative and not qualitative in nature.

Reporting for specific materials, systems and methods

We require information from authors about some types of materials, experimental systems and methods used in many studies. Here, indicate whether each material, system or method listed is relevant to your study. If you are not sure if a list item applies to your research, read the appropriate section before selecting a response.

Materials & experimental systems

n/a	Involved in the study	
[ ]	[x]	Antibodies
[x]	[ ]	Eukaryotic cell lines
[x]	[ ]	Palaeontology and archaeology
[ ]	[x]	Animals and other organisms
[x]	[ ]	Human research participants
[x]	[ ]	Clinical data
[x]	[ ]	Dual use research of concern

Methods

n/a	Involved in the study	
[x]	[ ]	ChIP-seq
[x]	[ ]	Flow cytometry
[x]	[ ]	MRI-based neuroimaging

Antibodies

Antibodies used	anti-DTk (Gp2), custom made<br>anti-CRY (R1), custom made<br>anti-CLK (Gp2), custom made<br>anti-PDF (C7), DSHB, AB_760350<br>anti-NC82, DSHB, AB_2314866<br>anti-PER (Rb1), custom made<br>anti-TIM (Rb1), custom made<br>anti-GFP, MBL international, 598<br>anti-RFP, MBL international, PM005
Validation	Validation of anti-DTk antibody was provided in this manuscript. Other custom made antibodies such as CRY, CLK, PER, and TIM had been validated in our own previous publications. anti-PDF and anti-NC82 antibodies have been used by a large number of laboratories including our own.

Animals and other organisms

Policy information about studies involving animals; ARRIVE guidelines recommended for reporting animal research

Laboratory animals	Drosophila melanogaster
Wild animals	n/a
Field-collected samples	n/a

Ethics oversight	No ethical approval was required because animals used were fruit flies.

Note that full information on the approval of the study protocol must also be provided in the manuscript.
